# Assessing the Efficacy of the INTELLECT Cognitive Behavioral Therapy Mobile App for Anxiety and Depressive Symptoms Among At-Risk Japanese Employees: Randomized Controlled Trial

**DOI:** 10.2196/60871

**Published:** 2025-06-24

**Authors:** Kengo Yokomitsu, Riki Oimatsu, Sean Han Yang Toh, Oliver Sündermann

**Affiliations:** 1 School of Psychological Sciences, University of Human Environments, 9-12, Dougo-himata, Matsuyama, Ehime, Japan, 81 899267007; 2 Intellect Japan Co, Ltd, Tokyo, Japan; 3 Department of Psychology, Harvard University, Cambridge, MA, United States; 4 Intellect Pte Ltd, Singapore, Singapore

**Keywords:** depressive symptom, internet cognitive behavioral therapy, randomized controlled trial, depression, behavior, cognitive, mobile app, anxiety, Japan, mobile health, employee, linear mixed model, questionnaire, feasibility, acceptability, self-monitoring usability

## Abstract

**Background:**

In Japan, the prevalence of anxiety and depressive symptoms within the working population has risen. This has been accentuated by the economic repercussions of the COVID-19 pandemic and the social isolation resulting from remote work setups. Mobile health apps, particularly those incorporating cognitive behavioral therapy (CBT) features, have shown potential in addressing these symptoms. These self-guided CBT interventions hold promise in alleviating the heightened depressive and anxiety symptoms often observed among Japanese employees.

**Objective:**

Using a randomized controlled trial, we compared the efficacy of the “INTELLECT” app against a no-treatment control group in improving depressive symptoms and CBT skills among Japanese full-time employees at postintervention and 2-month follow-up.

**Method:**

A total of 123 full-time Japanese employees were randomly allocated to either the intervention group (INTELLECT), where they engaged with self-help CBT features, or to a control group receiving no treatment. Intervention participants were required to engage with these features for at least 20 minutes per week over a span of 4 weeks. Weekly self-reported assessments were collected from all participants starting from baseline and continuing until the end of the 4-week intervention period. Subsequent assessments were conducted at 1-month and 2-month follow-up intervals. Linear mixed models were used to evaluate any effects of the self-guided intervention on depressive symptoms, as measured by the Patient Health Questionnaire-4, and cognitive behavioral skills, as measured by the Cognitive Behavioral Therapy Skills Scale. The app’s feasibility, usability, and acceptability ratings were also examined using the Implementation Outcome Scales for Digital Mental Health (iOSDMH).

**Results:**

The final sample (n=73) consisted of 46 (63%) participants who were female, 23 (32%) participants who were male, and 4 (6%) participants who identified as other genders, with a mean age of 40.4 (SD 10.7) years. Significant time × group interactions were found at postintervention and 2-month follow-up, with the intervention group (n=34) reporting significantly lower depressive symptoms than the control group (n=38) at postintervention (*t*_364.7426_=−2.243; *P*=.03; Cohen *d*=−0.57, 95% CI −1.07 to −0.06) and 2-month follow-up (*t*_364.6948_=−3.284; *P*<.001; Cohen *d*=−0.85, 95% CI −1.38 to −0.32). In addition, intervention participants reported significantly greater improvements in self-monitoring cognitive skills than control participants at postintervention (*t*_120.7526_=2.672; *P*=.01; Cohen *d*=0.68, 95% CI 0.17 to 1.18) but not follow-up (*t*_121.5475_=1.947; *P*=.05; Cohen *d*=0.50, 95% CI −0.01 to 1.02).

**Conclusions:**

This study provides evidence that CBT features on the INTELLECT app are effective in improving depressive symptoms and self-monitoring cognitive skills.

## Introduction

### Overview

Depressive and anxiety disorders wield substantial global influence. The Global Burden of Diseases, Injuries, and Risk Factors Study, for instance, identified depression and anxiety disorders as 2 of the most disabling mental disorders, ranking among the top 25 leading causes of global burden in 2019 [1]. The COVID-19 pandemic has also seen a notable surge of 27.6% in major depressive disorder cases and a 25.6% increase in anxiety disorder cases in 2020 [2]. Similar figures were reported in Japan, where a previous study [3] conducted a nationwide survey of over 8000 Japanese citizens and found that approximately one-fifth of respondents experienced clinically severe anxiety and depressive symptoms. Numerous studies also found that significant changes in the economic and working conditions caused by the pandemic contributed to a marked increase in symptoms of anxiety and depression among Japanese employees [4-6].

Despite the increasing prevalence of these symptoms, many Japanese employees primarily rely on informal counseling with family members or non-evidence-based self-management strategies for coping [7]. Concurrently, there is a growing demand for alternative, accessible mental health care options. Mobile health (mHealth) apps hold significant promise in delivering cost-effective, evidence-based solutions for mental disorders and in facilitating outcome monitoring. These services are especially valuable for socially disadvantaged groups, such as individuals who face barriers in accessing face-to-face clinical services [8]. Such services are also timely given the increasing ownership of mobile devices [9,10], which has facilitated access to mental health care in daily life, overcoming geographical and temporal limitations [11-14]. The increase in mental health apps in Japan is evident from a systematic review conducted between June 4 and June 11, 2021, which identified 172 mental health apps available on Japan’s Google Play Store and Apple App Store [15]. By September 2022, 53% of Japanese smartphone users were engaging with health care apps, indicating a remarkable 21% point increase from September 2019 [16]. The popularity of digital health care solutions seems to offer the potential for digital interventions to alleviate the declining mental health of Japanese employees. Several studies [17,18] have implemented digital interventions targeting Japanese workers and have reported reductions in depressive symptoms. However, these studies have primarily focused on employees with subthreshold depressive symptoms, and research involving higher-risk Japanese working populations remains limited. In Japan, digital mental health interventions are predominantly implemented in universally accessible formats designed to accommodate a broad spectrum of users. Prior research on such universally designed digital interventions has primarily concentrated on the prevention of major depressive episodes [19] or targeted individuals exhibiting subthreshold depressive symptoms [17,18]. Given the relatively high psychological barriers to seeking psychiatric services in Japan, there is substantial interest in anonymously accessible self-help interventions. Accordingly, evaluating the efficacy of universally designed programs in alleviating depressive symptoms among high-risk workers in Japan holds particular significance, not only for guiding future clinical applications but also for contributing to advancing research in this domain.

Moreover, it is now well established in the field that the effectiveness of digital cognitive behavioral therapy (CBT) in reducing depressive symptoms is supported by a growing body of evidence. Indeed, the previous study [20] found significantly larger effect sizes for self-help interventions involving CBT principles in improving anxiety and depression levels compared with no-treatment control groups. In some studies, these self-help CBT features even demonstrated comparable effectiveness and adherence rates to traditional face-to-face therapy [21,22]. Additionally, these features have reduced depressive symptoms in Japanese adults [23]. Given the importance of incorporating behavior change mechanisms to promote positive health behaviors, CBT is regarded as a fundamental therapeutic component in the delivery of mHealth apps for individuals experiencing mental health challenges. In this context, Tudor-Sfetea et al [24] found that compared with non-CBT-based interventions, CBT-based digital interventions are associated with greater user engagement and sustained app use, both of which contributed to facilitating behavioral change. Furthermore, Lin et al [25] highlighted that psychological empowerment and well-being in their life are key elements that can enhance the effectiveness of CBT-based mHealth interventions. Building on this perspective, this study explores how user engagement is also observable among Japanese working adults using a CBT-based intervention. Despite some evidence of these CBT self-help features, little is known about their effectiveness for clinically at-risk working adults with depressive and anxiety symptoms in Asia.

### This Study

The primary objective of the study is to examine whether self-help CBT-based features can improve depressive and anxiety symptoms among Japanese full-time employees experiencing mild, moderate, or severe levels of these symptoms. In selecting evidence-based features, the study uses “INTELLECT,” a mental health app that provides free access to a variety of evidence-based self-help features aimed at enhancing mental well-being for employees and consumers across Asia-Pacific countries. The efficacies for some of these features have been demonstrated in previous randomized controlled trials (RCTs) [26-28] and longitudinal studies [29,30]. In particular, the CBT-based features within INTELLECT have shown significant improvements in positive (ie, psychological resilience, body image perceptions, and self-compassion) [27,29] and negative mental well-being (ie, symptoms of anxiety and stress) [26,28] when compared with active control groups among working adults. However, no studies to our knowledge have tested the effectiveness of these features in clinically at-risk working populations. In an RCT setting, our primary hypothesis posits that the CBT features within the INTELLECT app will demonstrate greater efficacy in reducing symptoms of anxiety and depression among Japanese employees experiencing mild, moderate, or severe levels of these symptoms, compared with a waitlist control group. Our secondary objectives encompass evaluating whether these features also lead to improvements in CBT skills and assessing the acceptability, feasibility, and satisfaction levels among its users.

## Methods

### Trial Design

This study was an RCT with two groups: (1) a group engaging with self-help CBT features on an INTELLECT app for 3 weeks (intervention group) and (2) a no-treatment and assessment-only (AO) group (control group).

Outcome measures were assessed from baseline and continued until the end of the 4-week intervention period, with subsequent assessments conducted at 1-month and 2-month follow-up intervals. The primary dependent variable measured was depression level, while secondary dependent variables included cognitive behavioral skills. The app’s feasibility, usability, and acceptability ratings were also assessed using the Implementation Outcome Scales for Digital Mental Health (iOSDMH).

### Ethical Considerations

Ethics approval was given to the institutional review board of the first author’s affiliation (Ethics Review Committee, School of Psychological Sciences, University of Human Environments: 2023D-001). This trial was registered with the University Hospital Medical Information Network (UMIN) Center on June 16, 2023 (registration number: UMIN000051354). All participants provided informed consent before beginning the study. Consent was obtained electronically through a secure Google Survey platform after participants reviewed the study information sheet. Participation was entirely voluntary, and individuals could withdraw at any time without consequence. All data collected were anonymized prior to analysis. Personally identifiable information was neither collected nor stored. Data were stored on encrypted servers with restricted access to protect participant privacy and confidentiality.

Participants received compensation for their participation in the form of Amazon Gift Cards totaling ¥9000 Japanese yen (approximately US $64). Reimbursements were distributed in 3 stages: baseline (¥1000; approximately US $7), postintervention (¥3000; approximately US $21), and 2-month follow-up (¥5000; approximately US $36). All currency conversions to US dollars are based on the exchange rate at the time of the study (¥1=US $0.00713, as of June 16, 2023).

### Participants and Recruitment

Participants were included in the trial if they met the following inclusion criteria: (1) mild to severe depressive symptoms on the Japanese version of Patient Health Questionnaire-9 (PHQ-9) [31] with scores between 10 and 27, (2) aged 18 years or older, and (3) owning and able to use a smartphone/tablet device.

Participants were initially introduced to the study through web-based advertisements disseminated from June 16, 2023, to October 31, 2023. These advertisements directed potential participants to recruitment sites where participation information sheets and informed consent were provided. A total of 203 participants initially responded to online advertisements for recruitment. Of these, 130 were excluded from the study due to various reasons, including voluntary withdrawal, failure to complete the questionnaire, or not meeting the study’s inclusion criteria. The final sample (n=73) included 46 (63%) female participants, 23 (32%) male participants, and 4 (6%) participants identifying as other genders. The participants had a mean age of 40.4 (SD 10.7) years and were all full-time Japanese employees who owned a smartphone. Participants received monetary reimbursement for their participation.

Shortly after the trial began, the eligibility criteria were amended to eliminate the exclusion criterion related to suicidal ideation as assessed by item 9 of the PHQ-9. This revision was necessitated by the fact that all initial applicants were being excluded under this criterion, thereby rendering the continuation of the trial infeasible.

### Procedure

Participants who were directed to a dedicated recruitment website via online advertisements followed a link to a secured Google Survey, where participants completed baseline measures on the severity of depression (PHQ-9), and demographic information such as gender, age, occupation, education level, annual income, marital status, types of smartphones owned, and history of treatment for depression and structured psychotherapy for mental disorders to establish baseline ratings. Only full-time Japanese employees aged 18‐65 years with over mild levels of depressive symptoms (n=123) were then automatically randomized to one of 2 conditions—intervention (n=59) or active waitlist control (n=62)—using a computer-generated random sequence. Upon consenting to participate in the study, participants completed the primary outcome measure on the severity of depression (PHQ-4) and the secondary outcome measures on the skills related to cognitive behavioral therapy (Cognitive Behavioral Therapy Skills Scale [CBTSS]). In this study, they were not informed about the conditions they were allocated to and the real nature of the study. Instead, they were informed that the study would examine the efficacy of the mHealth app (INTELLECT) in promoting well-being.

Participants in the intervention group were then directed to download the INTELLECT mHealth app and provided with a unique code to register and access a research version of the app containing only the assigned program. This step was taken to ensure that any observed treatment effects were solely attributable to the program, rather than other features available in the app. It was verified whether participants had downloaded and registered the app through the backend system. Intervention group participants engaged with the “INTELLECT” app for a minimum of 20 minutes per week over a 4-week period, while active waitlist control group participants accessed the app once the intervention participants had completed the 4-week intervention. Engagement of the app for the intervention group was monitored on a weekly basis, and participants were informed when the app engagement was low. Five participants dropped out of the research, either due to low engagement with the app or loss of contact. Additionally, one participant’s data will not be included in the analysis due to excessive use of the app after 4 weeks.

Both intervention and control participants completed a series of self-report measures at 7 timepoints: baseline (PHQ-4, CBT-SS), week 1 (PHQ-4), week 2 (PHQ-4), week 3 (PHQ-4), postintervention (PHQ-4, CBT-SS, iOSDMH), 1-month (PHQ-4), and 2-month follow-up (PHQ-4, CBT-SS). Upon full participation, each participant received 9000 Japanese yen (approximately US $58) in Amazon Gift Cards as a token of appreciation. Reimbursements were distributed at 3 different timepoints as follows: Baseline (¥1000 Japanese yen [approximately US $6] Amazon Gift Card), Postintervention (¥3000 Japanese yen [approximately US $18] Amazon Gift Card), 2-month follow-up (¥3000 Japanese yen [approximately US $18] Amazon Gift Card).

### Intervention

INTELLECT is a consumer-based mental health app that offers users access to a variety of self-guided, evidence-based features, some of which have been validated in previous RCTs [26-28] and longitudinal studies [29,30]. The app’s “Home” tab provides access to 4 main self-care features as follows: “Learning Paths,” “Rescue Sessions,” “Wellbeing Check-in,” and “Guided Journals.” “Learning Paths” are designed to educate participants using evidence-based content to deepen their understanding and improve their self-management of mental health. For example, the “Feeling Depressed Learning Path” offers psychoeducation on cognitive distortions linked to low mood and depression, helping users learn how to reframe their thoughts. In other studies, this intervention has been shown to effectively aid users in altering their negative thoughts and emotions. “Rescue Sessions” are self-guided interventions that focus on addressing specific themes of adversity commonly faced by individuals. These themes include “overthinking,” “motivation,” “stress management,” “burnout,” and “relationships”. Each session provides targeted support using CBT-based strategies to overcome these challenges. The interventions are firmly rooted in CBT principles to identify and change negative thought patterns and behaviors. “Rescue Sessions” provides a comprehensive toolkit that enables individuals to strengthen their mental resilience and well-being by selecting the session most relevant to their current struggles, mindfulness, self-compassion, and CBT. The “Wellbeing Check-in” feature allows users to evaluate their feelings and stress levels by selecting emojis that best represent their current state. This daily self-monitoring tool encourages participants to track their emotions, fostering greater self-awareness and providing opportunities to manage negative emotions. “Guided Journals” offer various themes such as “reflection,” “problem-solving,” “goal-setting,” “sleep,” or “self-esteem.” Each journal prompts participants to write entries related to these themes, aiding in the identification and restructuring of cognitive patterns. For example, the “gratitude” journal encourages users to identify and record positive instances of the day, challenging momentary negative thought patterns and promoting cognitive restructuring skills. This practice helps foster a habit of considering alternative perspectives.

### Randomization, Allocation Concealment, and Blinding

Regarding assignment to both participant groups, INTELLECT Japan’s researcher conducted simple randomization using a computer-generated random sequence developed by the researcher. No blocking strategy was used during the allocation process. Stratification was used for the previous management of depression and structured psychotherapy for mental disorders. To implement the random allocation sequence, a roster of 73 individuals who consented to participate in the study was allocated to either the INTELLECT or AO groups according to the pregenerated random sequence described above. Additionally, all outcome measures were collected through self-administered online questionnaires, and the data collection process was designed such that individual participant responses could not influence the behavior or decisions of the study staff. These procedures were implemented to minimize bias during group allocation. However, complete allocation concealment may not have been fully ensured due to the potential predictability of the assignment sequence. Furthermore, owing to the nature of the study design, no efforts were made to match the intervention and control conditions in terms of appearance or delivery. Participants in the intervention group were granted immediate access to the app, whereas those assigned to the waitlist control group received no intervention during the trial period. Consequently, the 2 groups differed in both the content they received and their level of engagement. To minimize expectancy effects, all participants were informed that access to the app would be provided either immediately or following the completion of the study.

### Measures

No changes were made to the primary or secondary outcomes after the trial commenced.

#### Primary Outcome Measure

The Japanese version of Patient Health Questionnaire-4 (PHQ-4) [32] is a 4-item self-reported questionnaire for depression symptoms. Items are scored on a 4-point Likert scale (ranging from 0=“not at all” to 3=“nearly every day”), with higher scores indicating more depressive symptoms. In an earlier study [31], this scale demonstrated good internal consistency (α=.93) and good convergent validity (correlation coefficient with the Kessler Psychological Distress Scale: *r*=0.81) in a Japanese clinical population.

#### Secondary Outcome Measures

The CBTSS is a 32-items self-reported questionnaire that assesses the frequency of CBT skills utilization [33]. The CBTSS was included in this study to examine whether using the INTELLECT app improves cognitive behavioral therapy skills. Items are scored on a 4-point Likert-type scale (ranging from 1=“almost none of the above” to 4=“quite true”), with higher scores indicating frequent CBT skills utilization. This scale has a 5-factor structure (cognitive restructuring, behavioral activation, assertiveness communication, self-monitoring, and problem-solving). The high reliability and validity of this scale have been reported in previous studies involving Japanese workforce populations [33].

The Implementation Outcome Scales for Digital Mental Health (iOSDMH measures the feasibility, appropriateness, and acceptability of digital mental health interventions. This is a 19-items self-report measure using a 4-point Likert-type scale (ranging from 1=“disagree” to 4=“agree”). Internal consistency and validity were confirmed in the scale development study [34].

### Analytic Approach

Descriptive statistics were reported as means and SDs for continuous variables and frequencies for dichotomous or categorical variables. We used linear mixed modeling (LMM) to analyze the study’s primary outcome measures. LMM was selected because of its strength in accommodating missing data and its ability to incorporate random effects into analyses. In the primary analysis, the dependent variable was the PHQ-4 score, and the independent variables were assignment (categorical variables: INTELLECT, AO) and time (categorical variables: baseline [T1], and each follow-up [T2-T7]), with the interaction of assignment and time as a fixed-effect variable and participants as a random-effects variable. Secondary outcomes (secondary outcome measures) were analyzed in the same manner as the primary outcome. Moreover, the standardized effect sizes and 95% CI were estimated. Cohen *d* was the effect size reported, whereby 0.20 to 0.49 indicates a small effect, 0.50 to 0.79 indicates a moderate effect, and 0.80 indicates a large effect. For all analyses, *P*<.05 was considered statistically significant. All statistical analyses were performed using R software (version 4.0.2; R Foundation for Statistical Computing). The “lme4” package was used for LMM.

### Sample Size

In this study, no formal sample size calculation was performed prior to trial initiation due to the exploratory nature of the research. Recruitment was discontinued after enrolling 73 participants owing to research funding constraints. No interim analyses were planned or conducted, and no stopping guidelines were established.

## Results

### Participant Flow, Characteristics, and Recruitment

**Figure 1.**
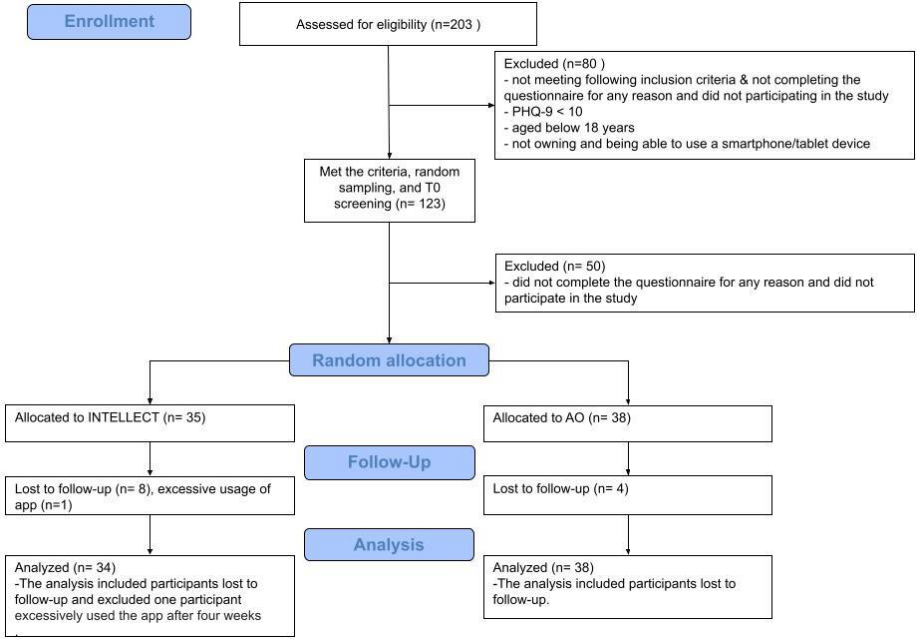
CONSORT (Consolidated Standards of Reporting Trails) flow diagram. AO: assessment-only; PHQ: Patient Health Questionnaire.

Of the 73 participants, 48% (n=35, mean age 38.91, SD 10.03 years) and 52% (n=38, mean age 41.82, SD 10.80 years) were assigned to INTELLECT and AO groups, respectively. Of the 35 participants allocated to the INTELLECT group, 8 were nonresponsive at the 2-month follow-up, as were 4 of the 38 participants in the AO group (Figure 1). The study period spanned between June 16, 2023, and March 31, 2024. Table 1 shows participants’ demographic data.

**Table 1.** Participant demographics and clinical characteristics (n=73).

	Total (n=73), n (%)	Intervention group (n=35), n (%)	Control group (n=38), n (%)
Gender			
Men	23 (31.5)	13 (37.1)	10 (26.3)
Women	46 (63.0)	20 (57.1)	26 (68.4)
Other	3 (4.1)	1 (2.9)	2 (5.3)
No response	1 (1.4)	1 (2.9)	0 (0)
Age (years)	40.42 (10.7)	38.91 (10.03)	41.82 (10.80)
Occupation			
Managing post	4 (5.5)	2 (5.7)	2 (5.3)
Technical job	16 (21.9)	6 (17.1)	10 (26.3)
Clerical worker	11 (15.1)	6 (17.1)	5 (13.2)
Sales staff	3 (4.1)	2 (5.7)	1 (2.6)
Service industry	15 (20.5)	9 (25.7)	6 (15.8)
Security worker	1 (1.4)	1 (2.9)	0 (0)
Production process worker	1 (1.4)	0 (0)	1 (2.6)
Transport and machine operation worker	3 (4.1)	1 (2.9)	2 (5.3)
Construction and mining worker	3 (4.1)	2 (57.0)	1 (2.6)
Carrying, cleaning, packaging, and related worker	2 (2.7)	0 (0)	2 (5.3)
Full-time homemaker	5 (6.8)	2 (5.7)	3 (7.9)
Family care/own illness treatment	3 (4.1)	2 (5.7)	1 (2.6)
Temporary retirement	5 (6.8)	2 (5.7)	3 (7.9)
Others	1 (4.1)	0 (0)	1 (2.6)
Education			
Elementary and junior high school	7 (9.6)	3 (8.6)	4 (10.5)
High school (equivalent test)	23 (31.5)	10 (28.6)	13 (34.2)
2-year and career college	8 (11.0)	6 (17.1)	2 (5.3)
4-year college	31 (42.5)	13 (37.1)	18 (47.4)
Graduate school	4 (5.5)	3 (8.6)	1 (2.6)
Income (monthly) (Japanese ¥)^a^			
<100,000	9 (12.3)	4 (11.4)	5 (13.2)
100,000‐199,999	11 (15.1)	4 (11.4)	7 (18.4)
200,000‐299,999	9 (12.3)	7 (20.0)	2 (5.3)
300,000‐399,999	23 (31.5)	10 (28.6)	13 (34.2)
400,000‐499,999	7 (9.6)	4 (11.4)	3 (7.9)
500,000‐599,999	6 (8.2)	3 (8.6)	3 (7.9)
600,000‐699,999	2 (2.7)	1 (2.9)	1 (2.6)
700,000‐799,999	3 (4.1)	1 (2.9)	2 (5.3)
800,000‐899,999	1 (1.4)	1 (2.9)	0 (0)
900,000‐999,999	1 (1.4)	0 (0)	1 (2.6)
1000,000‐1500,000	0 (0)	0 (0)	0 (0)
>1500,000	1 (1.4)	0 (0)	1 (2.6)
Marital status			
Married	33 (45.2)	15 (42.9)	18 (47.4)
Single	30 (41.1)	17 (48.6)	13 (34.2)
Divorced	10 (13.7)	3 (8.6)	7 (18.4)
Smartphone they are using			
Android	25 (34.2)	14 (40.0)	11 (28.9)
iPhone	46 (63.0)	20 (57.1)	26 (68.4)
Others	2 (2.7)	1 (2.9)	1 (2.6)
Previous management of depression	37 (50.7)	19 (54.3)	18 (47.4)
Previous structured psychotherapy for mental disorders other than depression	6 (8.2)	3 (8.6)	3 (7.9)
Habitual use of alcohol	8 (11.0)	6 (17.1)	2 (5.3)
Habitual use of tobacco	16 (21.9)	7 (20.0)	9 (23.7)

a ¥1=US $0.00713, as of June 16, 2023.

### Group Differences for the Primary Outcome Variable

72 participants were included in the analysis. The results showed that participants’ PHQ-4 scores significantly decreased from baseline at both postintervention and 2-month follow-up. There was a significant interaction between group and time at postintervention (*t*_364.7426_=−2.243, *P*=.03) and 2-month follow-up (*t*_364.6948_=−3.284, *P*<.001). As shown in Table 2, the effect sizes were moderate and large at postintervention and follow-up, respectively at postintervention (Cohen *d*=−0.57, 95% CI −1.07 to −0.06) and at 2 month follow-up (Cohen *d*=−0.85, 95% CI −1.38 to −0.32). A post hoc power analysis was conducted for the primary outcome based on a sample size of 72, an observed effect size of Cohen *d*=0.85, and a significance level of 0.05. The analysis indicated a power of approximately 99%, suggesting the study had sufficient statistical power to detect the observed effect.

**Table 2.** Results of depressive symptoms.

Group	Participants, n	Mean (SD)	Cohen *d*	95% CI
Time 1			–0.07	–0.53 to 0.39
Intervention	34	8.18 (3.19)		
Control	38	8.39 (2.97)		
Time 2			–0.19	–0.69 to 0.31
Intervention	30	7.23 (3.35)		
Control	33	8.00 (2.73)		
Time 3			–0.30	–0.81 to 0.22
Intervention	26	6.15 (3.71)		
Control	34	7.53 (3.09)		
Time 4			–0.14	–0.64 to 0.37
Intervention	27	6.52 (3.65)		
Control	34	6.97 (2.97)		
Time 5			–0.57	–1.07 to –0.06
Intervention	29	5.62 (3.53)		
Control	34	7.74 (3.12)		
Time 6			–0.57	–1.09 to –0.04
Intervention	26	4.62 (3.71)		
Control	33	6.91 (3.39)		
Time 7			–0.85	–1.38 to –0.32
Intervention	26	4.35 (2.37)		
Control	35	7.03 (3.55)		

### Group Differences for the Secondary Variable

As shown in Table 3, the results showed that participants’ CBTSS scores increased postintervention and follow-up. The intervention group showed a significant improvement in CBTSS’s self-monitoring score only at postintervention compared with the control group (*t*_120.7526_=2.672, *P*=.01). The effect size at postintervention was moderate (Cohen *d*=0.68, 95% CI 0.17 to 1.18). However, the intervention participants did not significantly improve self-monitoring cognitive skills than control participants at follow-up (*t*_121.5475_=1.947, *P*=.05, Cohen *d*=0.50, 95% CI −0.05 to 1.02).

**Table 3.** Results of the Cognitive Behavioral Therapy Skills Scale scores.

Group	Time 1				Time 2				Time 3			
	Participants, n	Mean (SD)	Cohen *d*	95% CI	Participants, n	Mean (SD)	Cohen *d*	95% CI	Participants, n	Mean (SD)	Cohen *d*	95% CI
Cognitive restructuring			–0.08	–0.54 to 0.38			0.42	–0.08 to 0.92			0.42	–0.09 to 0.93
Intervention	34	6.18 (4.37)			29	8.79 (4.69)			26	8.92 (4.22)		
Control	38	6.50 (3.52)			34	7.26 (4.21)			35	7.43 (4.19)		
Behavioral activation			0.08	–0.39 to 0.54			0.49	–0.02 to 0.99			0.32	–0.19 to 0.83
Intervention	34	4.85 (3.17)			29	7.07 (3.70)			26	7.69 (3.67)		
Control	38	4.58 (3.75)			34	4.71 (3.91)			35	6.09 (4.28)		
Assertiveness communication			0.09	–0.37 to 0.55			0.23	–0.27 to 0.72			0.25	–0.26 to 0.76
Intervention	34	9.15 (3.65)			29	10.21 (3.52)			26	10.92 (3.38)		
Control	38	8.84 (3.37)			34	9.35 (3.61)			35	9.80 (3.01)		
Self-monitoring			–0.13	–0.59 to 0.34			0.68	0.17 to 1.18			0.50	–0.05 to 1.02
Intervention	34	7.35 (4.55)			29	11.97 (4.85)			26	11.81 (4.17)		
Control	38	7.89 (4.09)			34	8.76 (4.06)			35	9.51 (5.06)		
Problem solving			0.24	–0.23 to 0.70			0.34	–0.16 to 0.83			0.48	–0.03 to 1.00
Intervention	34	6.65 (3.99)			29	8.83 (3.86)			26	9.81 (4.25)		
Control	38	5.76 (3.50)			34	6.56 (3.74)			35	6.86 (4.07)		

### App Engagement Among the Intervention Group

As shown in Table 4, the average degree of satisfaction measured by the iOSDMH among users of the INTELLECT app was notably high. Among the 29 intervention participants, 16‐24 (55%‐83%) participants answered “agree” or “relatively agree” on each item of acceptability (n=16‐24, 55%‐83%), appropriateness (n=19‐24, 66%‐83%), and feasibility (n=7‐23, 24%‐79%). The highest proportions of positive responses in each of the 3 aspects were “ this program is acceptable for me”(24 out of 29 participants, 83%) in acceptability, “the content of the program is appropriate” (24 out of 29 participants, 83%) in appropriateness, and “the length of 1 content is implementable; the program is easy to understand” (23 out of 29 participants, 79%) in feasibility, respectively. Regarding overall satisfaction with INTELLECT app, 23 out of 29 participants (79%) answered “agree” or “relatively agree.” Regarding harms, 2‐8 (7%‐28%) participants answered “agree” or “relatively agree,” except for “excessive pressure on learning this program regularly” (12 out of 29 participants, 41%). Over the 4-week period, the “Wellbeing Check-in” module was the most frequently used, followed by “Rescue Sessions” and “Guided Journals.” Notably, the “Learning Path” component required the most time investment from users, likely explaining the discrepancy between its number of initial engagements and lower completion rate.

**Table 4.** Results of the INTELLECT app engagement.

	Mean (SD)	Zeroth quartile	First quartile	Median	Third quartile	Fourth quartile
iOSDMH^a^						
Acceptability	8.59 (2.40)	3.00	6.50	9.00	10.00	12.00
Appropriateness	11.59 (2.61)	4.00	10.50	12.00	13.00	16.00
Feasibility	16.52 (2.85)	10.00	15.00	17.00	18.00	21.00
Satisfaction	2.86 (0.74)	1.00	3.00	3.00	3.00	4.00
Harms	8.52 (3.30)	5.00	6.00	8.00	10.00	18.00
Used components						
Starting learning paths	5.19 (3.03)	2	3	4	5	13
Completed learning paths	3.63 (2.72)	1	2	3	4	11
Rescue sessions	7.67 (5.28)	0	4	6	11	20
Wellbeing check-in	15.11 (8.31)	1	9	15	21	33
Guided journals	7.19 (5.68)	1	2	6	11	22

a iOSDMH: Implementation Outcome Scales for Digital Mental Health.

## Discussion

### Principal Results

The primary aim of this study was to compare the efficacy of INTELLECT’s CBT-based features in improving anxiety and depressive symptoms among Japanese employees experiencing mild, moderate, or severe levels of these symptoms, compared with a waitlist control group. In addition, the study examined whether these features resulted in significant improvements in CBT-related life skills and assessed the acceptability, feasibility, and satisfaction levels among users.

Our primary hypothesis was supported, with the intervention group showing significantly greater improvements in depression and anxiety levels at postintervention and 2-month follow-up. Effect sizes of these improvements at each timepoint were considerably larger than those reported in similar studies reviewed by a recent meta-analysis [35]. These findings add to the scant literature by showing that self-guided CBT features can be highly effective in reducing anxiety and depressive symptoms among clinically at-risk working adults in Japan. This aligns with previous research showing comparable efficacy of brief CBT-based mobile features, or computerized CBT in alleviating depressive symptoms among university students experiencing baseline depressive symptoms in Western societies [36-38].

Our secondary hypothesis was partially supported. The self-guided CBT features also demonstrated significantly greater improvements in the self-monitoring aspect of cognitive behavioral skills than the waitlist control group, as assessed through the CBTSS. The effect size of this improvement at postintervention was moderate (Cohen *d*=0.68, 95% CI 0.17 to 1.18). Self-monitoring questions are prominently featured throughout INTELLECT’s self-guided features. For example, thought records are embedded within all of INTELLECT’s “Learning Paths,” “Rescue Sessions”, and “Guided Journals,” consistently prompting each participant to analyze their own thought patterns. It is probable that the decrease in anxiety and depressive symptoms among our participants was related to the increase in self-monitoring. This technique alone has previously shown efficacy in alleviating anxiety and depressive symptoms in adults [39,40] and youths diagnosed with major depression [41]. However, our study did not conduct longitudinal mediation analyses to confirm this hypothesis. Future research could use more rigorous statistical methods, such as multilevel mediation analyses, to support this claim. In contrast, for other skills assessed by the CBTSS (ie, Behavioral Activation, Assertiveness Communication, Problem Solving), only specific self-guided features within INTELLECT were tailored to enhance these skills. For example, within INTELLECT’s suite of Learning Paths, approximately half of them concentrate on modifying behavioral patterns, while only 3 focus on modifying communication styles. Researchers have also proposed that the anticipated type of improvements resulting from the interventions correlate with the type of content offered within the intervention [42]. Future researchers are encouraged to isolate various types of self-guided interventions and assess the efficacy of each intervention using a similar RCT design to ours. In comparison to the control group, the intervention group also did not exhibit significantly greater improvements in the cognitive restructuring aspect of CBT skills. There are a couple of possible reasons. First, cognitive restructuring has been identified as one of the most challenging CBT skills to impart, even among trained counselors and psychotherapists with many years of experience [43]. Furthermore, cognitive restructuring is widely recognized as a difficult skill to master independently, both within and outside of therapeutic contexts [44,45]. Further research is needed to explore suitable methodologies for effectively teaching cognitive restructuring skills within self-help modalities, enabling users to learn autonomously. Finally, it is important to note that participants in both the intervention and control groups demonstrated significant improvements in CBT skills over time (Table 3). This particular control group may have masked the intervention effects on CBT skills. Future researchers can more effectively assess the efficacy of INTELLECT’s self-guided CBT interventions by using a 3-armed RCT, wherein the efficacy of these CBT features is compared against those of an active control group and a waitlist control group.

Finally, it is noteworthy that a sizable percentage of our participants expressed satisfaction with using INTELLECT, rating it highly in terms of acceptability, appropriateness for improving mental health, and ease of understanding. The self-help features of INTELLECT, which use plain language, illustrations, videos, and audio narration to explain evidence-based content, may contribute to these positive implementation outcomes. Given the substantial effect sizes and user-friendly design of this app, we recommend broader dissemination of the INTELLECT app to alleviate anxiety and depressive symptoms among employees in need. Lin et al [25] highlighted that psychological empowerment and inspiration are key elements that can enhance the effectiveness of CBT-based mHealth interventions. Future studies must conduct a more detailed analysis of the role of user engagement in enhancing the effectiveness of CBT-based interventions.

### Limitation

This study has 3 limitations. First, although INTELLECT’s self-help interventions significantly reduced depressive symptoms during the study period and at the 2-month follow-up, it is uncertain if these improvements would persist beyond 2 months. Previous research on similar internet-based CBT interventions suggests a potential decline in efficacy over extended durations. Hence, it is important for future studies to evaluate the longitudinal effects of mobile-based CBT interventions. Second, one methodological limitation of this study concerns the potential predictability of the group assignment sequence. Group allocation was conducted using simple randomization based on a computer-generated sequence developed in advance by the researcher, without the use of blocking. Although stratification was applied for participants’ history of depression management and structured psychotherapy, and the researcher had no access to other participant data, the fixed nature of the sequence may have allowed for some degree of predictability. Consequently, full allocation concealment could not be ensured. While there is no indication that this affected the integrity of the assignment process, this limitation should be taken into account when interpreting the internal validity of the study findings. Third, this intervention study primarily focused on assessing improvements in depressive symptoms and user engagement with the app, overlooking potential adverse effects associated with its use. A thorough examination of postintervention side effects is essential to ensure the app’s safety across diverse user demographics. Last, while the INTELLECT app effectively enhanced self-monitoring capabilities, it did not significantly influence the development of other crucial CBT-related skills, such as cognitive restructuring, behavioral activation, communication, or problem-solving. Future studies may explore strategies for enhancing the app to facilitate a more comprehensive development of these skills, which could augment INTELLECT’s efficacy in addressing anxiety and depressive symptoms.

### Conclusions

This study provides the first evidence of the efficacy of the INTELLECT app, including all components, in reducing depressive symptoms among Japanese adult workers. The use of the INTELLECT app significantly enhanced self-monitoring skills, a key component of the observed improvement. However, the absence of postintervention effects on other CBT skills underscores the necessity for further investigation in these areas.

## Supplementary material

10.2196/60871 Checklist 1 CONSORT-eHEALTH checklist (V 1.6.1). CONSORT: Consolidated Standards of Reporting Trails.

## Abbreviations

AO: assessment-only
CBT: cognitive behavioral therapy
CBTSS: Cognitive Behavioral Therapy Skills Scale
iOSDMH: Implementation Outcome Scales for Digital Mental Health
LMM: linear mixed model
mHealth: mobile health
PHQ: Patient Health Questionnaire
RCT: randomized controlled trial
UMIN: University Hospital Medical Information Network
