# Supplementary material for: Assessing the Efficacy of the INTELLECT Cognitive Behavioral Therapy Mobile App for Anxiety and Depressive Symptoms Among At-Risk Japanese Employees: Randomized Controlled Trial
Source: JMIR Mhealth Uhealth. 2025 Jun 24;13:e60871. doi: 10.2196/60871 (PMC12212887; doi:10.2196/60871)
Supplement: Checklist 1 [file mhealth-v13-e60871-s001.pdf]

CONSORT-EHEALTH (V 1.6.1) - Submission/Publication Form

The CONSORT-EHEALTH checklist is intended for authors of randomized trials evaluating web-based and Internet-based applications/interventions, including mobile interventions, electronic games (incl multiplayer games), social media, certain telehealth applications, and other interactive and/or networked electronic applications. Some of the items (e.g. all subitems under item 5 - description of the intervention) may also be applicable for other study designs.

The goal of the CONSORT EHEALTH checklist and guideline is to be
a) a guide for reporting for authors of RCTs,
b) to form a basis for appraisal of an ehealth trial (in terms of validity)

CONSORT-EHEALTH items/subitems are MANDATORY reporting items for studies published in the Journal of Medical Internet Research and other journals / scientific societies endorsing the checklist.

Items numbered 1., 2., 3., 4a., 4b etc are original CONSORT or CONSORT-NPT (non-pharmacologic treatment) items.
Items with Roman numerals (i., ii, iii, iv etc.) are CONSORT-EHEALTH extensions/clarifications.

As the CONSORT-EHEALTH checklist is still considered in a formative stage, we would ask that you also RATE ON A SCALE OF 1-5 how important/useful you feel each item is FOR THE PURPOSE OF THE CHECKLIST and reporting guideline (optional).

Mandatory reporting items are marked with a red *.
In the textboxes, either copy & paste the relevant sections from your manuscript into this form - please include any quotes from your manuscript in QUOTATION MARKS,
or answer directly by providing additional information not in the manuscript, or elaborating on why the item was not relevant for this study.

YOUR ANSWERS WILL BE PUBLISHED AS A SUPPLEMENTARY FILE TO YOUR PUBLICATION IN JMIR AND ARE CONSIDERED PART OF YOUR PUBLICATION (IF ACCEPTED).
Please fill in these questions diligently. Information will not be copyedited, so please use proper spelling and grammar, use correct capitalization, and avoid abbreviations.

DO NOT FORGET TO SAVE AS PDF _AND_ CLICK THE SUBMIT BUTTON SO YOUR ANSWERS ARE IN OUR DATABASE !!!

Citation Suggestion (if you append the pdf as Appendix we suggest to cite this paper in the caption):
Eysenbach G, CONSORT-EHEALTH Group
[illegible]

回答のサイズが大きすぎます。一部の回答を短くしてみてください。

[illegible]

URL: http://www.jmir.org/2011/4/e126/
doi: 10.2196/jmir.1923
PMID: 22209829

yokomitsuken5@gmail.com アカウントを切り替える

共有なし

* 必須の質問です

Your name *

First Last

Kengo Yokomitsu

Primary Affiliation (short), City, Country *

University of Toronto, Toronto, Canada

University of Human Environments, Ehime, Jap

Your e-mail address *

abc@gmail.com

yokomitsuken5@gmail.com

Title of your manuscript *

Provide the (draft) title of your manuscript.

Randomized controlled trial of a Cognitive-Behavioural Therapy Mobile Application (INTELLECT) in alleviating anxiety and depressive symptoms among Japanese employees with depressive symptoms

回答のサイズが大きすぎます。一部の回答を短くしてみてください。

Name of your App/Software/Intervention *

If there is a short and a long/alternate name, write the short name first and add the long name in brackets.

the INTELLECT app

Evaluated Version (if any)

e.g. "V1", "Release 2017-03-01", "Version 2.0.27913"

version 4.6.2v10

Language(s) *

What language is the intervention/app in? If multiple languages are available, separate by comma (e.g. "English, French")

Japanese, English

URL of your Intervention Website or App

e.g. a direct link to the mobile app on app in appstore (itunes, Google Play), or URL of the website. If the intervention is a DVD or hardware, you can also link to an Amazon page.

https://apps.apple.com/jp/app/intellect-create-a-better-you/id1483308512

URL of an image/screenshot (optional)

https://drive.google.com/drive/folders/1tI2shG51derGbCuxzNg2RjoPDxlyue9u

回答のサイズが大きすぎます。一部の回答を短くしてみてください。

Accessibility *

Can an enduser access the intervention presently?

- ○ access is free and open
- ○ access only for special usergroups, not open
- ◉ access is open to everyone, but requires payment/subscription/in-app purchases
- ○ app/intervention no longer accessible
- ○ その他:

Primary Medical Indication/Disease/Condition *

e.g. "Stress", "Diabetes", or define the target group in brackets after the condition, e.g. "Autism (Parents of children with)", "Alzheimers (Informal Caregivers of)"

anxiety and depressive symptoms

Primary Outcomes measured in trial *

comma-separated list of primary outcomes reported in the trial

PHQ-4 (anxiety and depressive symptoms)

Secondary/other outcomes

Are there any other outcomes the intervention is expected to affect?

CBTSS (the skills related to cognitive behavioural therapy)

回答のサイズが大きすぎます。一部の回答を短くしてみてください。

Recommended "Dose" *

What do the instructions for users say on how often the app should be used?

- (●) Approximately Daily
- ( ) Approximately Weekly
- ( ) Approximately Monthly
- ( ) Approximately Yearly
- ( ) "as needed"
- ( ) その他:

Approx. Percentage of Users (starters) still using the app as recommended after 3 months *

- (●) unknown / not evaluated
- ( ) 0-10%
- ( ) 11-20%
- ( ) 21-30%
- ( ) 31-40%
- ( ) 41-50%
- ( ) 51-60%
- ( ) 61-70%
- ( ) 71%-80%
- ( ) 81-90%
- ( ) 91-100%
- ( ) その他:

回答のサイズが大きすぎます。一部の回答を短くしてみてください。

Overall, was the app/intervention effective? *

- (●) yes: all primary outcomes were significantly better in intervention group vs control
- ( ) partly: SOME primary outcomes were significantly better in intervention group vs control
- ( ) no statistically significant difference between control and intervention
- ( ) potentially harmful: control was significantly better than intervention in one or more outcomes
- ( ) inconclusive: more research is needed
- ( ) その他:

Article Preparation Status/Stage *

At which stage in your article preparation are you currently (at the time you fill in this form)

- ( ) not submitted yet - in early draft status
- ( ) not submitted yet - in late draft status, just before submission
- (●) submitted to a journal but not reviewed yet
- ( ) submitted to a journal and after receiving initial reviewer comments
- ( ) submitted to a journal and accepted, but not published yet
- ( ) published
- ( ) その他:

回答のサイズが大きすぎます。一部の回答を短くしてみてください。

Journal *

If you already know where you will submit this paper (or if it is already submitted), please provide the journal name (if it is not JMIR, provide the journal name under "other")

- ○ not submitted yet / unclear where I will submit this
- ○ Journal of Medical Internet Research (JMIR)
- ◉ JMIR mHealth and UHealth
- ○ JMIR Serious Games
- ○ JMIR Mental Health
- ○ JMIR Public Health
- ○ JMIR Formative Research
- ○ Other JMIR sister journal
- ○ その他:

Is this a full powered effectiveness trial or a pilot/feasibility trial? *

- ◉ Pilot/feasibility
- ○ Fully powered

Manuscript tracking number *

If this is a JMIR submission, please provide the manuscript tracking number under "other" (The ms tracking number can be found in the submission acknowledgement email, or when you login as author in JMIR. If the paper is already published in JMIR, then the ms tracking number is the four-digit number at the end of the DOI, to be found at the bottom of each published article in JMIR)

- ○ no ms number (yet) / not (yet) submitted to / published in JMIR
- ◉ その他: #60871

回答のサイズが大きすぎます。一部の回答を短くしてみてください。

TITLE AND ABSTRACT

1a) TITLE: Identification as a randomized trial in the title

1a) Does your paper address CONSORT item 1a? *

I.e does the title contain the phrase "Randomized Controlled Trial"? (if not, explain the reason under "other")

- (selected) yes
- その他:

1a-i) Identify the mode of delivery in the title

Identify the mode of delivery. Preferably use “web-based” and/or “mobile” and/or “electronic game” in the title. Avoid ambiguous terms like “online”, “virtual”, “interactive”. Use “Internet-based” only if Intervention includes non-web-based Internet components (e.g. email), use “computer-based” or “electronic” only if offline products are used. Use “virtual” only in the context of “virtual reality” (3-D worlds). Use “online” only in the context of “online support groups”. Complement or substitute product names with broader terms for the class of products (such as “mobile” or “smart phone” instead of “iphone”), especially if the application runs on different platforms.

	1	2	3	4	5	
subitem not at all important	○	○	○	○	●	essential

選択を解除

回答のサイズが大きすぎます。一部の回答を短くしてみてください。

Does your paper address subitem 1a-i? *

Copy and paste relevant sections from manuscript title (include quotes in quotation marks "like this" to indicate direct quotes from your manuscript), or elaborate on this item by providing additional information not in the ms, or briefly explain why the item is not applicable/relevant for your study

Randomized controlled trial of a Cognitive-Behavioural Therapy "Mobile" Application (INTELLECT) in alleviating anxiety and depressive symptoms among Japanese employees with depressive symptoms

1a-ii) Non-web-based components or important co-interventions in title

Mention non-web-based components or important co-interventions in title, if any (e.g., “with telephone support”).

	1	2	3	4	5	
subitem not at all important	◉	○	○	○	○	essential

選択を解除

Does your paper address subitem 1a-ii?

Copy and paste relevant sections from manuscript title (include quotes in quotation marks "like this" to indicate direct quotes from your manuscript), or elaborate on this item by providing additional information not in the ms, or briefly explain why the item is not applicable/relevant for your study

回答を入力

回答のサイズが大きすぎます。一部の回答を短くしてみてください。

1a-iii) Primary condition or target group in the title

Mention primary condition or target group in the title, if any (e.g., “for children with Type I Diabetes”) Example: A Web-based and Mobile Intervention with Telephone Support for Children with Type I Diabetes: Randomized Controlled Trial

	1	2	3	4	5	
subitem not at all important	○	○	○	○	◉	essential

選択を解除

Does your paper address subitem 1a-iii? *

Copy and paste relevant sections from manuscript title (include quotes in quotation marks "like this" to indicate direct quotes from your manuscript), or elaborate on this item by providing additional information not in the ms, or briefly explain why the item is not applicable/relevant for your study

Randomized controlled trial of a Cognitive-Behavioural Therapy Mobile Application (INTELLECT) in alleviating "anxiety and depressive symptoms among Japanese employees with depressive symptoms"

1b) ABSTRACT: Structured summary of trial design, methods, results, and conclusions

NPT extension: Description of experimental treatment, comparator, care providers, centers, and blinding status.

回答のサイズが大きすぎます。一部の回答を短くしてみてください。

1b-i) Key features/functionalities/components of the intervention and comparator in the METHODS section of the ABSTRACT

Mention key features/functionalities/components of the intervention and comparator in the abstract. If possible, also mention theories and principles used for designing the site. Keep in mind the needs of systematic reviewers and indexers by including important synonyms. (Note: Only report in the abstract what the main paper is reporting. If this information is missing from the main body of text, consider adding it)

	1	2	3	4	5	
subitem not at all important	○	○	○	○	◉	essential

選択を解除

Does your paper address subitem 1b-i? *

Copy and paste relevant sections from the manuscript abstract (include quotes in quotation marks "like this" to indicate direct quotes from your manuscript), or elaborate on this item by providing additional information not in the ms, or briefly explain why the item is not applicable/relevant for your study

where they engaged with self-help CBT features, or to "a control group receiving no treatment".

1b-ii) Level of human involvement in the METHODS section of the ABSTRACT

Clarify the level of human involvement in the abstract, e.g., use phrases like “fully automated” vs. “therapist/nurse/care provider/physician-assisted” (mention number and expertise of providers involved, if any). (Note: Only report in the abstract what the main paper is reporting. If this information is missing from the main body of text, consider adding it)

	1	2	3	4	5	
subitem not at all important	○	○	○	◉	○	essential

選択を解除

回答のサイズが大きすぎます。一部の回答を短くしてみてください。

Does your paper address subitem 1b-ii?

Copy and paste relevant sections from the manuscript abstract (include quotes in quotation marks "like this" to indicate direct quotes from your manuscript), or elaborate on this item by providing additional information not in the ms, or briefly explain why the item is not applicable/relevant for your study

they engaged with "self-help" CBT features

1b-iii) Open vs. closed, web-based (self-assessment) vs. face-to-face assessments in the METHODS section of the ABSTRACT

Mention how participants were recruited (online vs. offline), e.g., from an open access website or from a clinic or a closed online user group (closed usergroup trial), and clarify if this was a purely web-based trial, or there were face-to-face components (as part of the intervention or for assessment). Clearly say if outcomes were self-assessed through questionnaires (as common in web-based trials). Note: In traditional offline trials, an open trial (open-label trial) is a type of clinical trial in which both the researchers and participants know which treatment is being administered. To avoid confusion, use “blinded” or “unblinded” to indicated the level of blinding instead of “open”, as “open” in web-based trials usually refers to “open access” (i.e. participants can self-enrol). (Note: Only report in the abstract what the main paper is reporting. If this information is missing from the main body of text, consider adding it)

	1	2	3	4	5	
subitem not at all important	○	●	○	○	○	essential

選択を解除

Does your paper address subitem 1b-iii?

Copy and paste relevant sections from the manuscript abstract (include quotes in quotation marks "like this" to indicate direct quotes from your manuscript), or elaborate on this item by providing additional information not in the ms, or briefly explain why the item is not applicable/relevant for your study

回答を入力

回答のサイズが大きすぎます。一部の回答を短くしてみてください。

1b-iv) RESULTS section in abstract must contain use data

Report number of participants enrolled/assessed in each group, the use/uptake of the intervention (e.g., attrition/adherence metrics, use over time, number of logins etc.), in addition to primary/secondary outcomes. (Note: Only report in the abstract what the main paper is reporting. If this information is missing from the main body of text, consider adding it)

	1	2	3	4	5	
subitem not at all important	○	○	○	○	◉	essential

選択を解除

Does your paper address subitem 1b-iv?

Copy and paste relevant sections from the manuscript abstract (include quotes in quotation marks "like this" to indicate direct quotes from your manuscript), or elaborate on this item by providing additional information not in the ms, or briefly explain why the item is not applicable/relevant for your study

"The final sample (n=73) consisted of 46 (63.0%) female participants, 23 (31.5%) male participants, and 4 (5.5%) participants identifying as other genders, with a mean age of 40.4 (SD=10.7) years. Significant time x group interactions were found at post-intervention and 2-month follow-up, with the intervention group (n=34) reporting significantly lower depressive symptoms than the control group (n=38) at post-intervention (Cohen’s d=-0.57 [-1.07, -0.06]) and 2-month follow-up (Cohen’s d=-0.85 [-1.38, -0.32]). In addition, intervention participants reported significantly greater improvements in self-monitoring cognitive skills than control participants at post-intervention (Cohen’s d=0.68 [0.17, 1.18]) but not follow-up (Cohen’s d=0.50 [-0.05, 1.02]). "

回答のサイズが大きすぎます。一部の回答を短くしてみてください。

1b-v) CONCLUSIONS/DISCUSSION in abstract for negative trials

Conclusions/Discussions in abstract for negative trials: Discuss the primary outcome - if the trial is negative (primary outcome not changed), and the intervention was not used, discuss whether negative results are attributable to lack of uptake and discuss reasons. (Note: Only report in the abstract what the main paper is reporting. If this information is missing from the main body of text, consider adding it)

	1	2	3	4	5	
subitem not at all important	○	○	○	○	◉	essential

選択を解除

Does your paper address subitem 1b-v?

Copy and paste relevant sections from the manuscript abstract (include quotes in quotation marks "like this" to indicate direct quotes from your manuscript), or elaborate on this item by providing additional information not in the ms, or briefly explain why the item is not applicable/relevant for your study

”This study provides evidence that CBT features on the INTELLECT app are effective in improving depressive symptoms and self-monitoring cognitive skills. ”

”Despite some evidence of these CBT self-help features, little is known about their effectiveness for clinically at-risk working adults with depressive and anxiety symptoms in Asia.”

"However, no studies to our knowledge have tested the effectiveness of these features in clinically at-risk working populations."

INTRODUCTION

2a) In INTRODUCTION: Scientific background and explanation of rationale

回答のサイズが大きすぎます。一部の回答を短くしてみてください。

2a-i) Problem and the type of system/solution

Describe the problem and the type of system/solution that is object of the study: intended as stand-alone intervention vs. incorporated in broader health care program? Intended for a particular patient population? Goals of the intervention, e.g., being more cost-effective to other interventions, replace or complement other solutions? (Note: Details about the intervention are provided in “Methods” under 5)

	1	2	3	4	5	
subitem not at all important	○	○	○	◉	○	essential

選択を解除

Does your paper address subitem 2a-i? *

Copy and paste relevant sections from the manuscript (include quotes in quotation marks "like this" to indicate direct quotes from your manuscript), or elaborate on this item by providing additional information not in the ms, or briefly explain why the item is not applicable/relevant for your study

”The popularity of digital healthcare solutions seems to offer the potential for digital interventions to alleviate the declining mental health of Japanese employees.”

2a-ii) Scientific background, rationale: What is known about the (type of) system

Scientific background, rationale: What is known about the (type of) system that is the object of the study (be sure to discuss the use of similar systems for other conditions/diagnoses, if appropiate), motivation for the study, i.e. what are the reasons for and what is the context for this specific study, from which stakeholder viewpoint is the study performed, potential impact of findings [2]. Briefly justify the choice of the comparator.

	1	2	3	4	5	
subitem not at all important	○	○	○	○	◉	essential

選択を解除

回答のサイズが大きすぎます。一部の回答を短くしてみてください。

Does your paper address subitem 2a-ii? *

Copy and paste relevant sections from the manuscript (include quotes in quotation marks "like this" to indicate direct quotes from your manuscript), or elaborate on this item by providing additional information not in the ms, or briefly explain why the item is not applicable/relevant for your study

About self-hep CBT

”Indeed, Linardon et al. (2019) found significantly larger effect sizes for self-help interventions involving Cognitive Behavioral Therapy (CBT) principles in improving anxiety and depression levels compared to no-treatment control groups. In some studies, these self-help CBT features even demonstrated comparable effectiveness and adherence rates to traditional face-to-face therapy (Carlbring et al., 2018; van Ballegooijen et al., 2014). Additionally, these features have reduced depressive symptoms in Japanese adults (Karyotaki et al., 2017). ”

About our INTELLECT app

”In selecting evidence-based features, the study utilizes “INTELLECT”, a mental health application that provides free access to a variety of evidence-based self-help features aimed at enhancing mental well-being for employees and consumers across Asia-Pacific countries. The efficacies for some of these features have been demonstrated in previous randomized-controlled trials (RCT) (Kosasih et al., 2023; Ong et al., 2022; Toh et al., 2022) and longitudinal studies (Toh et al., 2023a; Toh et al., 2023b). In particular, the CBT-based features within INTELLECT have shown significant improvements in positive (i.e., psychological resilience, body image perceptions, self-compassion) (Ong et al., 2022; Toh et al., 2023) and negative mental well-being (i.e., symptoms of anxiety and stress) (Toh et al., 2022; Kosasih et al., 2023) when compared to active control groups among working adults. ”

2b) In INTRODUCTION: Specific objectives or hypotheses

回答のサイズが大きすぎます。一部の回答を短くしてみてください。

Does your paper address CONSORT subitem 2b? *

Copy and paste relevant sections from the manuscript (include quotes in quotation marks "like this" to indicate direct quotes from your manuscript), or elaborate on this item by providing additional information not in the ms, or briefly explain why the item is not applicable/relevant for your study

Objective

"The primary objective of the study is to examine whether self-help CBT-based features can improve depressive and anxiety symptoms among Japanese full-time employees experiencing mild, moderate, or severe levels of these symptoms."

Hypothesis

"In an RCT setting, our primary hypothesis posits that the CBT features within the INTELLECT app will demonstrate greater efficacy in reducing symptoms of anxiety and depression among Japanese employees experiencing mild, moderate, or severe levels of these symptoms, compared to a waitlist control group. Our secondary objectives encompass evaluating whether these features also lead to improvements in cognitive behavioral therapy skills and assessing the acceptability, feasibility, and satisfaction levels among its users. "

METHODS

3a) Description of trial design (such as parallel, factorial) including allocation ratio

Does your paper address CONSORT subitem 3a? *

Copy and paste relevant sections from the manuscript (include quotes in quotation marks "like this" to indicate direct quotes from your manuscript), or elaborate on this item by providing additional information not in the ms, or briefly explain why the item is not applicable/relevant for your study

Trail Design
”This study was a RCT with two groups: (1) a group engaging with self-help CBT features on an intellect app for three weeks (intervention group) and (2) a group receiving no treatment and assessment- only group (control group). ”

回答のサイズが大きすぎます。一部の回答を短くしてみてください。

3b) Important changes to methods after trial commencement (such as eligibility criteria), with reasons

Does your paper address CONSORT subitem 3b? *

Copy and paste relevant sections from the manuscript (include quotes in quotation marks "like this" to indicate direct quotes from your manuscript), or elaborate on this item by providing additional information not in the ms, or briefly explain why the item is not applicable/relevant for your study

We do not have important changes to methods after trial commencement

3b-i) Bug fixes, Downtimes, Content Changes

Bug fixes, Downtimes, Content Changes: ehealth systems are often dynamic systems. A description of changes to methods therefore also includes important changes made on the intervention or comparator during the trial (e.g., major bug fixes or changes in the functionality or content) (5-iii) and other "unexpected events" that may have influenced study design such as staff changes, system failures/downtimes, etc. [2].

	1	2	3	4	5	
subitem not at all important	◉	○	○	○	○	essential

選択を解除

Does your paper address subitem 3b-i?

Copy and paste relevant sections from the manuscript (include quotes in quotation marks "like this" to indicate direct quotes from your manuscript), or elaborate on this item by providing additional information not in the ms, or briefly explain why the item is not applicable/relevant for your study

Our study do not have Bug fixes, Downtimes, Content Changes

4a) Eligibility criteria for participants

回答のサイズが大きすぎます。一部の回答を短くしてみてください。

Does your paper address CONSORT subitem 4a? *

Copy and paste relevant sections from the manuscript (include quotes in quotation marks "like this" to indicate direct quotes from your manuscript), or elaborate on this item by providing additional information not in the ms, or briefly explain why the item is not applicable/relevant for your study

Figure 1

"Excluded
- not meeting following inclusion criteria & not completing the questionnaire for any reason and did not participating in the study
- PHQ-9 < 10
- age of 18 years before
- not owning and being able to use a smartphone/tablet device"

4a-i) Computer / Internet literacy

Computer / Internet literacy is often an implicit “de facto” eligibility criterion - this should be explicitly clarified.

	1	2	3	4	5	
subitem not at all important	○	○	○	○	◉	essential

選択を解除

Does your paper address subitem 4a-i?

Copy and paste relevant sections from the manuscript (include quotes in quotation marks "like this" to indicate direct quotes from your manuscript), or elaborate on this item by providing additional information not in the ms, or briefly explain why the item is not applicable/relevant for your study

Figure 1

Excluded
- not meeting following inclusion criteria & not completing the questionnaire for any reason and did not participating in the study
- PHQ-9 < 10
- age of 18 years before
- [illegible]

回答のサイズが大きすぎます。一部の回答を短くしてみてください。

4a-ii) Open vs. closed, web-based vs. face-to-face assessments:

Open vs. closed, web-based vs. face-to-face assessments: Mention how participants were recruited (online vs. offline), e.g., from an open access website or from a clinic, and clarify if this was a purely web-based trial, or there were face-to-face components (as part of the intervention or for assessment), i.e., to what degree got the study team to know the participant. In online-only trials, clarify if participants were quasi-anonymous and whether having multiple identities was possible or whether technical or logistical measures (e.g., cookies, email confirmation, phone calls) were used to detect/prevent these.

	1	2	3	4	5	
subitem not at all important	○	○	○	○	◉	essential

選択を解除

Does your paper address subitem 4a-ii? *

Copy and paste relevant sections from the manuscript (include quotes in quotation marks "like this" to indicate direct quotes from your manuscript), or elaborate on this item by providing additional information not in the ms, or briefly explain why the item is not applicable/relevant for your study

”Participants were initially introduced to the study through online advertisements disseminated from June 16th 2023 to October 31st 2023. These advertisements directed potential participants to recruitment sites where participation information sheets and informed consent were provided. Subsequently, participants followed a link to a secured Google Survey, where participants completed baseline measures on the severity of depression (PHQ-9), various psychiatric symptoms (CCS), and demographic information such as gender, age, occupation, education level, annual income, marital status, types of smartphones owned, and history of treatment for depression and structured psychotherapy for mental disorders to establish baseline ratings. ”

回答のサイズが大きすぎます。一部の回答を短くしてみてください。

4a-iii) Information giving during recruitment

Information given during recruitment. Specify how participants were briefed for recruitment and in the informed consent procedures (e.g., publish the informed consent documentation as appendix, see also item X26), as this information may have an effect on user self-selection, user expectation and may also bias results.

	1	2	3	4	5	
subitem not at all important	○	○	◉	○	○	essential

選択を解除

Does your paper address subitem 4a-iii?

Copy and paste relevant sections from the manuscript (include quotes in quotation marks "like this" to indicate direct quotes from your manuscript), or elaborate on this item by providing additional information not in the ms, or briefly explain why the item is not applicable/relevant for your study

Procedure
Participants were initially introduced to the study through online advertisements disseminated from June 16th 2023 to October 31st 2023. These advertisements directed potential participants to recruitment sites where participation information sheets and ”informed consent were provided”.

”In this study, they were not informed about the conditions they were allocated to and the real nature of the study. Instead, they were informed that the study would examine the efficacy of the mHealth app (Intellect) in promoting well-being.”

4b) Settings and locations where the data were collected

回答のサイズが大きすぎます。一部の回答を短くしてみてください。

Does your paper address CONSORT subitem 4b? *

Copy and paste relevant sections from the manuscript (include quotes in quotation marks "like this" to indicate direct quotes from your manuscript), or elaborate on this item by providing additional information not in the ms, or briefly explain why the item is not applicable/relevant for your study

"Both intervention and control participants completed a series of self-report measures at seven timepoints: baseline (PHQ-4, CBT-SS), Week 1 (PHQ-4), Week 2 (PHQ-4), Week 3 (PHQ-4), post-intervention (PHQ-4, CBT-SS, iOSDMH), 1-month (PHQ-4), and 2-month follow-up (PHQ-4, CBT-SS). "

4b-i) Report if outcomes were (self-)assessed through online questionnaires

Clearly report if outcomes were (self-)assessed through online questionnaires (as common in web-based trials) or otherwise.

	1	2	3	4	5	
subitem not at all important	○	○	◉	○	○	essential

選択を解除

Does your paper address subitem 4b-i? *

Copy and paste relevant sections from the manuscript (include quotes in quotation marks "like this" to indicate direct quotes from your manuscript), or elaborate on this item by providing additional information not in the ms, or briefly explain why the item is not applicable/relevant for your study

"participants followed a link to a secured Google Survey", where participants completed baseline measures on the severity of depression (PHQ-9), various psychiatric symptoms (CCS), and demographic information such as gender, age, occupation, education level, annual income, marital status, types of smartphones owned, and history of treatment for depression and structured psychotherapy for mental disorders to establish baseline ratings.

回答のサイズが大きすぎます。一部の回答を短くしてみてください。

4b-ii) Report how institutional affiliations are displayed

Report how institutional affiliations are displayed to potential participants [on ehealth media], as affiliations with prestigious hospitals or universities may affect volunteer rates, use, and reactions with regards to an intervention.(Not a required item – describe only if this may bias results)

	1	2	3	4	5	
subitem not at all important	◉	○	○	○	○	essential

選択を解除

Does your paper address subitem 4b-ii?

Copy and paste relevant sections from the manuscript (include quotes in quotation marks "like this" to indicate direct quotes from your manuscript), or elaborate on this item by providing additional information not in the ms, or briefly explain why the item is not applicable/relevant for your study

we do not report how institutional affiliation are displayed

5) The interventions for each group with sufficient details to allow replication, including how and when they were actually administered

5-i) Mention names, credential, affiliations of the developers, sponsors, and owners

Mention names, credential, affiliations of the developers, sponsors, and owners [6] (if authors/evaluators are owners or developer of the software, this needs to be declared in a "Conflict of interest" section or mentioned elsewhere in the manuscript).

	1	2	3	4	5	
subitem not at all important	○	○	◉	○	○	essential

選択を解除

回答のサイズが大きすぎます。一部の回答を短くしてみてください。

Does your paper address subitem 5-i?

Copy and paste relevant sections from the manuscript (include quotes in quotation marks "like this" to indicate direct quotes from your manuscript), or elaborate on this item by providing additional information not in the ms, or briefly explain why the item is not applicable/relevant for your study

Acknowledgements
This study was self-funded by Intellect Pte Ltd under a Research Collaboration Agreement with the University of Human Environments, Japan.
Authors’ Contributions

All of the authors contributed substantially to the conceptualisation of this study. KY and RO led the data collection procedures, data analysis, and wrote the initial draft of the manuscript. SHYT revised all sections of the manuscript and finalized the draft with the approval of KY. OS supervised the study’s procedures.

Conflicts of Interest
The study was fully funded by Intellect Pte Ltd. The study design, data management, interpretation, analysis, reporting, and decision to publish of the study are entirely independent of Intellect Pte Ltd.

5-ii) Describe the history/development process

Describe the history/development process of the application and previous formative evaluations (e.g., focus groups, usability testing), as these will have an impact on adoption/use rates and help with interpreting results.

	1	2	3	4	5	
subitem not at all important	○	○	○	◉	○	essential

選択を解除

回答のサイズが大きすぎます。一部の回答を短くしてみてください。

Does your paper address subitem 5-ii?

Copy and paste relevant sections from the manuscript (include quotes in quotation marks "like this" to indicate direct quotes from your manuscript), or elaborate on this item by providing additional information not in the ms, or briefly explain why the item is not applicable/relevant for your study

"Intervention
Intellect is a consumer-based mental health application that offers users access to a variety of self-guided, evidence-based features, some of which have been validated in previous randomized controlled trials (Kosasih et al., 2023; Ong et al., 2022; Toh et al., 2022) and longitudinal studies (Toh et al., 2023a; Toh et al., 2023b). "

5-iii) Revisions and updating

Revisions and updating. Clearly mention the date and/or version number of the application/intervention (and comparator, if applicable) evaluated, or describe whether the intervention underwent major changes during the evaluation process, or whether the development and/or content was “frozen” during the trial. Describe dynamic components such as news feeds or changing content which may have an impact on the replicability of the intervention (for unexpected events see item 3b).

	1	2	3	4	5	
subitem not at all important	◉	○	○	○	○	essential

選択を解除

Does your paper address subitem 5-iii?

Copy and paste relevant sections from the manuscript (include quotes in quotation marks "like this" to indicate direct quotes from your manuscript), or elaborate on this item by providing additional information not in the ms, or briefly explain why the item is not applicable/relevant for your study

we did not revise and update during our study

回答のサイズが大きすぎます。一部の回答を短くしてみてください。

5-iv) Quality assurance methods

Provide information on quality assurance methods to ensure accuracy and quality of information provided [1], if applicable.

	1	2	3	4	5	
subitem not at all important	◉	○	○	○	○	essential

選択を解除

Does your paper address subitem 5-iv?

Copy and paste relevant sections from the manuscript (include quotes in quotation marks "like this" to indicate direct quotes from your manuscript), or elaborate on this item by providing additional information not in the ms, or briefly explain why the item is not applicable/relevant for your study

This item is not applicable.

5-v) Ensure replicability by publishing the source code, and/or providing screenshots/screen-capture video, and/or providing flowcharts of the algorithms used

Ensure replicability by publishing the source code, and/or providing screenshots/screen-capture video, and/or providing flowcharts of the algorithms used. Replicability (i.e., other researchers should in principle be able to replicate the study) is a hallmark of scientific reporting.

	1	2	3	4	5	
subitem not at all important	○	○	○	○	◉	essential

選択を解除

回答のサイズが大きすぎます。一部の回答を短くしてみてください。

Does your paper address subitem 5-v?

Copy and paste relevant sections from the manuscript (include quotes in quotation marks "like this" to indicate direct quotes from your manuscript), or elaborate on this item by providing additional information not in the ms, or briefly explain why the item is not applicable/relevant for your study

”Images of these self-care features can be found in Appendix A.”

5-vi) Digital preservation

Digital preservation: Provide the URL of the application, but as the intervention is likely to change or disappear over the course of the years; also make sure the intervention is archived (Internet Archive, webcitation.org, and/or publishing the source code or screenshots/videos alongside the article). As pages behind login screens cannot be archived, consider creating demo pages which are accessible without login.

	1	2	3	4	5	
subitem not at all important	○	○	○	○	○	essential

Does your paper address subitem 5-vi?

Copy and paste relevant sections from the manuscript (include quotes in quotation marks "like this" to indicate direct quotes from your manuscript), or elaborate on this item by providing additional information not in the ms, or briefly explain why the item is not applicable/relevant for your study

https://apps.apple.com/jp/app/intellect-create-a-better-you/id1483308512, https://play.google.com/store/apps/details?id=co.intellect.app&hl=en_US

回答のサイズが大きすぎます。一部の回答を短くしてみてください。

5-vii) Access

Access: Describe how participants accessed the application, in what setting/context, if they had to pay (or were paid) or not, whether they had to be a member of specific group. If known, describe how participants obtained “access to the platform and Internet” [1]. To ensure access for editors/reviewers/readers, consider to provide a “backdoor” login account or demo mode for reviewers/readers to explore the application (also important for archiving purposes, see vi).

	1	2	3	4	5	
subitem not at all important	○	○	○	◉	○	essential

選択を解除

Does your paper address subitem 5-vii? *

Copy and paste relevant sections from the manuscript (include quotes in quotation marks "like this" to indicate direct quotes from your manuscript), or elaborate on this item by providing additional information not in the ms, or briefly explain why the item is not applicable/relevant for your study

"Participants in the intervention group were then directed to download the Intellect mHealth application and provided with a unique code to register and access a research version of the app containing only the assigned program. This step was taken to ensure that any observed treatment effects were solely attributable to the program, rather than other features available in the application. It was verified whether participants had downloaded and registered the app through the backend system. "

回答のサイズが大きすぎます。一部の回答を短くしてみてください。

5-viii) Mode of delivery, features/functionalities/components of the intervention and comparator, and the theoretical framework

Describe mode of delivery, features/functionalities/components of the intervention and comparator, and the theoretical framework [6] used to design them (instructional strategy [1], behaviour change techniques, persuasive features, etc., see e.g., [7, 8] for terminology). This includes an in-depth description of the content (including where it is coming from and who developed it) [1]," whether [and how] it is tailored to individual circumstances and allows users to track their progress and receive feedback" [6]. This also includes a description of communication delivery channels and – if computer-mediated communication is a component – whether communication was synchronous or asynchronous [6]. It also includes information on presentation strategies [1], including page design principles, average amount of text on pages, presence of hyperlinks to other resources, etc. [1].

	1	2	3	4	5	
subitem not at all important	○	○	○	○	◉	essential

選択を解除

回答のサイズが大きすぎます。一部の回答を短くしてみてください。

Does your paper address subitem 5-viii? *

Copy and paste relevant sections from the manuscript (include quotes in quotation marks "like this" to indicate direct quotes from your manuscript), or elaborate on this item by providing additional information not in the ms, or briefly explain why the item is not applicable/relevant for your study

Intellect is a consumer-based mental health application that offers users access to a variety of self-guided, evidence-based features, some of which have been validated in previous randomized controlled trials (Kosasih et al., 2023; Ong et al., 2022; Toh et al., 2022) and longitudinal studies (Toh et al., 2023a; Toh et al., 2023b).

"The app's 'Home' tab provides access to four main self-care features: 'Learning Paths', 'Rescue Sessions', 'Wellbeing Check-in', and 'Guided Journals'. "Learning Paths' are designed to educate participants using evidence-based content to deepen their understanding and improve their self-management of mental health. For example, the 'Feeling Depressed Learning Path' offers psychoeducation on cognitive distortions linked to low mood and depression, helping users learn how to reframe their thoughts. In other studies, this intervention has been shown to effectively aid users in altering their negative thoughts and emotions. 'Rescue Sessions' are self-guided interventions that focus on addressing specific themes of adversity commonly faced by individuals. These themes include 'overthinking', 'motivation', 'stress management', 'burnout', and 'relationships'. Each session provides targeted support using CBT-based strategies to overcome these challenges. The interventions are firmly rooted in CBT principles to identify and change negative thought patterns and behaviors. 'Rescue Sessions' provide a comprehensive toolkit that enables individuals to strengthen their mental resilience and well-being by selecting the session most relevant to their current struggles. mindfulness, self-compassion, and cognitive behavioral therapy The 'Wellbeing Check-in' feature allows users to evaluate their feelings and stress levels by selecting emojis that best represent their current state. This daily self-monitoring tool encourages participants to track their emotions, fostering greater self-awareness and providing opportunities to manage negative emotions. 'Guided Journals' offer various themes such as 'reflection', 'problem-solving', 'goal-setting', 'sleep', or 'self-esteem'. Each journal prompts participants to write entries related to these themes, aiding in the identification and restructuring of cognitive patterns. For example, the 'gratitude' journal encourages users to identify and record positive instances of the day, challenging momentary negative thought patterns and promoting cognitive restructuring skills. This practice helps foster a habit of considering alternative perspectives . . Images of these self-care features can be found in Appendix A."

回答のサイズが大きすぎます。一部の回答を短くしてみてください。

5-ix) Describe use parameters

Describe use parameters (e.g., intended “doses” and optimal timing for use). Clarify what instructions or recommendations were given to the user, e.g., regarding timing, frequency, heaviness of use, if any, or was the intervention used ad libitum.

	1	2	3	4	5	
subitem not at all important	○	○	○	◉	○	essential

選択を解除

Does your paper address subitem 5-ix?

Copy and paste relevant sections from the manuscript (include quotes in quotation marks "like this" to indicate direct quotes from your manuscript), or elaborate on this item by providing additional information not in the ms, or briefly explain why the item is not applicable/relevant for your study

"Intervention group participants engaged with the "Intellect" application for a minimum of 20 minutes per week over a 4-week period, while active wait-list control group participants accessed the application once the intervention participants had completed the 4-week intervention. "

5-x) Clarify the level of human involvement

Clarify the level of human involvement (care providers or health professionals, also technical assistance) in the e-intervention or as co-intervention (detail number and expertise of professionals involved, if any, as well as “type of assistance offered, the timing and frequency of the support, how it is initiated, and the medium by which the assistance is delivered”. It may be necessary to distinguish between the level of human involvement required for the trial, and the level of human involvement required for a routine application outside of a RCT setting (discuss under item 21 – generalizability).

	1	2	3	4	5	
subitem not at all important	○	○	○	◉	○	essential

選択を解除

回答のサイズが大きすぎます。一部の回答を短くしてみてください。

Does your paper address subitem 5-x?

Copy and paste relevant sections from the manuscript (include quotes in quotation marks "like this" to indicate direct quotes from your manuscript), or elaborate on this item by providing additional information not in the ms, or briefly explain why the item is not applicable/relevant for your study

"Engagement of the app for the intervention group was monitored on a weekly basis and participants were informed when the app engagement was low. Five participants dropped out of the research, either due to low engagement with the app or loss of contact. Additionally, one participant's data will not be included in the analysis due to excessive use of the app after four weeks."

5-xi) Report any prompts/reminders used

Report any prompts/reminders used: Clarify if there were prompts (letters, emails, phone calls, SMS) to use the application, what triggered them, frequency etc. It may be necessary to distinguish between the level of prompts/reminders required for the trial, and the level of prompts/reminders for a routine application outside of a RCT setting (discuss under item 21 – generalizability).

	1	2	3	4	5	
subitem not at all important	○	○	○	◉	○	essential

選択を解除

Does your paper address subitem 5-xi? *

Copy and paste relevant sections from the manuscript (include quotes in quotation marks "like this" to indicate direct quotes from your manuscript), or elaborate on this item by providing additional information not in the ms, or briefly explain why the item is not applicable/relevant for your study

"Engagement of the app for the intervention group was monitored on a weekly basis and participants were informed when the app engagement was low. Five participants dropped out of the research, either due to low engagement with the app or loss of contact. Additionally, one participant's data will not be included in the analysis due to excessive use of the app after four weeks."

回答のサイズが大きすぎます。一部の回答を短くしてみてください。

5-xii) Describe any co-interventions (incl. training/support)

Describe any co-interventions (incl. training/support): Clearly state any interventions that are provided in addition to the targeted eHealth intervention, as ehealth intervention may not be designed as stand-alone intervention. This includes training sessions and support [1]. It may be necessary to distinguish between the level of training required for the trial, and the level of training for a routine application outside of a RCT setting (discuss under item 21 – generalizability.

	1	2	3	4	5	
subitem not at all important	(●)	○	○	○	○	essential

選択を解除

Does your paper address subitem 5-xii? *

Copy and paste relevant sections from the manuscript (include quotes in quotation marks "like this" to indicate direct quotes from your manuscript), or elaborate on this item by providing additional information not in the ms, or briefly explain why the item is not applicable/relevant for your study

we do not have any co-interventions

6a) Completely defined pre-specified primary and secondary outcome measures, including how and when they were assessed

Does your paper address CONSORT subitem 6a? *

Copy and paste relevant sections from the manuscript (include quotes in quotation marks "like this" to indicate direct quotes from your manuscript), or elaborate on this item by providing additional information not in the ms, or briefly explain why the item is not applicable/relevant for your study

" The primary dependent variable measured was depression level while secondary dependent variables included cognitive behavioral skills."

回答のサイズが大きすぎます。一部の回答を短くしてみてください。

6a-i) Online questionnaires: describe if they were validated for online use and apply CHERRIES items to describe how the questionnaires were designed/deployed

If outcomes were obtained through online questionnaires, describe if they were validated for online use and apply CHERRIES items to describe how the questionnaires were designed/deployed [9].

	1	2	3	4	5	
subitem not at all important	○	○	◉	○	○	essential

選択を解除

Does your paper address subitem 6a-i?

Copy and paste relevant sections from manuscript text

"participants followed a link to a secured Google Survey", where participants completed baseline measures on the severity of depression (PHQ-9), various psychiatric symptoms (CCS), and demographic information such as gender, age, occupation, education level, annual income, marital status, types of smartphones owned, and history of treatment for depression and structured psychotherapy for mental disorders to establish baseline ratings.

6a-ii) Describe whether and how "use" (including intensity of use/dosage) was defined/measured/monitored

Describe whether and how "use" (including intensity of use/dosage) was defined/measured/monitored (logins, logfile analysis, etc.). Use/adoption metrics are important process outcomes that should be reported in any ehealth trial.

	1	2	3	4	5	
subitem not at all important	○	○	○	◉	○	essential

選択を解除

回答のサイズが大きすぎます。一部の回答を短くしてみてください。

Does your paper address subitem 6a-ii?

Copy and paste relevant sections from manuscript text

Intervention group participants engaged with the "Intellect" application for a minimum of 20 minutes per week over a 4-week period, while active wait-list control group participants accessed the application once the intervention participants had completed the 4-week intervention. "Engagement of the app for the intervention group was monitored on a weekly basis and participants were informed when the app engagement was low. Five participants dropped out of the research, either due to low engagement with the app or loss of contact. Additionally, one participant's data will not be included in the analysis due to excessive use of the app after four weeks. "

6a-iii) Describe whether, how, and when qualitative feedback from participants was obtained

Describe whether, how, and when qualitative feedback from participants was obtained (e.g., through emails, feedback forms, interviews, focus groups).

	1	2	3	4	5	
subitem not at all important	◉	○	○	○	○	essential

選択を解除

Does your paper address subitem 6a-iii?

Copy and paste relevant sections from manuscript text

we do not collect qualitative feedback from participants

6b) Any changes to trial outcomes after the trial commenced, with reasons

回答のサイズが大きすぎます。一部の回答を短くしてみてください。

Does your paper address CONSORT subitem 6b? *

Copy and paste relevant sections from the manuscript (include quotes in quotation marks "like this" to indicate direct quotes from your manuscript), or elaborate on this item by providing additional information not in the ms, or briefly explain why the item is not applicable/relevant for your study

we do not have any changes

7a) How sample size was determined

NPT: When applicable, details of whether and how the clustering by care provides or centers was addressed

7a-i) Describe whether and how expected attrition was taken into account when calculating the sample size

Describe whether and how expected attrition was taken into account when calculating the sample size.

	1	2	3	4	5	
subitem not at all important	◉	○	○	○	○	essential

選択を解除

Does your paper address subitem 7a-i?

Copy and paste relevant sections from manuscript title (include quotes in quotation marks "like this" to indicate direct quotes from your manuscript), or elaborate on this item by providing additional information not in the ms, or briefly explain why the item is not applicable/relevant for your study

回答を入力

7b) When applicable, explanation of any interim analyses and stopping guidelines

回答のサイズが大きすぎます。一部の回答を短くしてみてください。

Does your paper address CONSORT subitem 7b? *

Copy and paste relevant sections from the manuscript (include quotes in quotation marks "like this" to indicate direct quotes from your manuscript), or elaborate on this item by providing additional information not in the ms, or briefly explain why the item is not applicable/relevant for your study

”Intervention group participants engaged with the "Intellect" application for a minimum of 20 minutes per week over a 4-week period”

8a) Method used to generate the random allocation sequence

NPT: When applicable, how care providers were allocated to each trial group

Does your paper address CONSORT subitem 8a? *

Copy and paste relevant sections from the manuscript (include quotes in quotation marks "like this" to indicate direct quotes from your manuscript), or elaborate on this item by providing additional information not in the ms, or briefly explain why the item is not applicable/relevant for your study

”Regarding assignment to both participant groups, Intellect Japan’s researcher conducted simple randomization using a computer-generated random sequence developed by the researcher.

8b) Type of randomisation; details of any restriction (such as blocking and block size)

Does your paper address CONSORT subitem 8b? *

Copy and paste relevant sections from the manuscript (include quotes in quotation marks "like this" to indicate direct quotes from your manuscript), or elaborate on this item by providing additional information not in the ms, or briefly explain why the item is not applicable/relevant for your study

”No blocking strategy was employed during the allocation process. Stratification was employed for previous management of depression and structured psychotherapy for mental disorders.

回答のサイズが大きすぎます。一部の回答を短くしてみてください。

9) Mechanism used to implement the random allocation sequence (such as sequentially numbered containers), describing any steps taken to conceal the sequence until interventions were assigned

Does your paper address CONSORT subitem 9? *

Copy and paste relevant sections from the manuscript (include quotes in quotation marks "like this" to indicate direct quotes from your manuscript), or elaborate on this item by providing additional information not in the ms, or briefly explain why the item is not applicable/relevant for your study

”To implement the random allocation sequence, a roster of 73 individuals who consented to participate in the study were systematically and alternately assigned to either the INTELLECT or AO groups, commencing from the top of the list. For allocation concealment and blinding, the participants were provided a detailed explanation of the intervention, and the Intellect Japan’s researcher created the assignment without reviewing any screening, baseline, or other information about the participants behind previous management for depression and structured psychotherapy for mental disorders. ”

10) Who generated the random allocation sequence, who enrolled participants, and who assigned participants to interventions

回答のサイズが大きすぎます。一部の回答を短くしてみてください。

Does your paper address CONSORT subitem 10? *

Copy and paste relevant sections from the manuscript (include quotes in quotation marks "like this" to indicate direct quotes from your manuscript), or elaborate on this item by providing additional information not in the ms, or briefly explain why the item is not applicable/relevant for your study

Regarding assignment to both participant groups, Intellect Japan's researcher conducted simple randomization using a computer-generated random sequence developed by ”the researcher. ”

No blocking strategy was employed during the allocation process. Stratification was employed for previous management of depression and structured psychotherapy for mental disorders. To implement the random allocation sequence, a roster of 73 individuals who consented to participate in the study were systematically and alternately assigned to either the INTELLECT or AO groups, commencing from the top of the list. For allocation concealment and blinding, the participants were provided a detailed explanation of the intervention, and "the Intellect Japan's researcher" created the assignment without reviewing any screening, baseline, or other information about the participants behind previous management for depression and structured psychotherapy for mental disorders.

11a) If done, who was blinded after assignment to interventions (for example, participants, care providers, those assessing outcomes) and how

NPT: Whether or not administering co-interventions were blinded to group assignment

11a-i) Specify who was blinded, and who wasn't

Specify who was blinded, and who wasn't. Usually, in web-based trials it is not possible to blind the participants [1, 3] (this should be clearly acknowledged), but it may be possible to blind outcome assessors, those doing data analysis or those administering co-interventions (if any).

	1	2	3	4	5	
subitem not at all important	◉	○	○	○	○	essential

選択を解除

回答のサイズが大きすぎます。一部の回答を短くしてみてください。

Does your paper address subitem 11a-i? *

Copy and paste relevant sections from the manuscript (include quotes in quotation marks "like this" to indicate direct quotes from your manuscript), or elaborate on this item by providing additional information not in the ms, or briefly explain why the item is not applicable/relevant for your study

Blind was not conducted in our study.

11a-ii) Discuss e.g., whether participants knew which intervention was the “intervention of interest” and which one was the “comparator”

Informed consent procedures (4a-ii) can create biases and certain expectations - discuss e.g., whether participants knew which intervention was the “intervention of interest” and which one was the “comparator”.

	1	2	3	4	5	
subitem not at all important	○	○	○	○	◉	essential

選択を解除

Does your paper address subitem 11a-ii?

Copy and paste relevant sections from the manuscript (include quotes in quotation marks "like this" to indicate direct quotes from your manuscript), or elaborate on this item by providing additional information not in the ms, or briefly explain why the item is not applicable/relevant for your study

To implement the random allocation sequence, a roster of 73 individuals who consented to participate in the study were systematically and alternately assigned to either the INTELLECT or AO groups, commencing from the top of the list. For allocation concealment and blinding, "the participants were provided a detailed explanation of the intervention", and the Intellect Japan’s researcher created the assignment without reviewing any screening, baseline, or other information about the participants behind previous management for depression and structured psychotherapy for mental disorders.

11b) If relevant, description of the similarity of interventions

(this item is usually not relevant for ehealth trials as it refers to similarity of a placebo or [illegible]

回答のサイズが大きすぎます。一部の回答を短くしてみてください。

Does your paper address CONSORT subitem 11b? *

Copy and paste relevant sections from the manuscript (include quotes in quotation marks "like this" to indicate direct quotes from your manuscript), or elaborate on this item by providing additional information not in the ms, or briefly explain why the item is not applicable/relevant for your study

this is not applicable in this study

12a) Statistical methods used to compare groups for primary and secondary outcomes

NPT: When applicable, details of whether and how the clustering by care providers or centers was addressed

Does your paper address CONSORT subitem 12a? *

Copy and paste relevant sections from the manuscript (include quotes in quotation marks "like this" to indicate direct quotes from your manuscript), or elaborate on this item by providing additional information not in the ms, or briefly explain why the item is not applicable/relevant for your study

"We used linear mixed modelling (LMM) to analyze the study's primary outcome measures. LMM was selected because of its strength in accommodating missing data and its ability to incorporate random effects into analyses. In the primary analysis, the dependent variable was the PHQ-4 score, and the independent variables were assignment (categorical variables: INTELLECT, AO) and time (categorical variables: baseline [T1], and each follow-up [T2–T7]), with the interaction of assignment and time as a fixed-effect variable and participants as a random-effects variable. Secondary outcomes (secondary outcome measures) were analyzed in the same manner as the primary outcome."

回答のサイズが大きすぎます。一部の回答を短くしてみてください。

12a-i) Imputation techniques to deal with attrition / missing values

Imputation techniques to deal with attrition / missing values: Not all participants will use the intervention/comparator as intended and attrition is typically high in ehealth trials. Specify how participants who did not use the application or dropped out from the trial were treated in the statistical analysis (a complete case analysis is strongly discouraged, and simple imputation techniques such as LOCF may also be problematic [4]).

	1	2	3	4	5	
subitem not at all important	○	○	○	◉	○	essential

選択を解除

Does your paper address subitem 12a-i? *

Copy and paste relevant sections from the manuscript (include quotes in quotation marks "like this" to indicate direct quotes from your manuscript), or elaborate on this item by providing additional information not in the ms, or briefly explain why the item is not applicable/relevant for your study

We used linear mixed modelling (LMM) to analyze the study's primary outcome measures. LMM was selected because of its strength in "accommodating missing data" and its ability to incorporate random effects into analyses. In the primary analysis, the dependent variable was the PHQ-4 score, and the independent variables were assignment (categorical variables: INTELLECT, AO) and time (categorical variables: baseline [T1], and each follow-up [T2–T7]), with the interaction of assignment and time as a fixed-effect variable and participants as a random-effects variable. Secondary outcomes (secondary outcome measures) were analyzed in the same manner as the primary outcome.

12b) Methods for additional analyses, such as subgroup analyses and adjusted analyses

Does your paper address CONSORT subitem 12b? *

Copy and paste relevant sections from the manuscript (include quotes in quotation marks "like this" to indicate direct quotes from your manuscript), or elaborate on this item by providing additional information not in the ms, or briefly explain why the item is not applicable/relevant for your study

回答のサイズが大きすぎます。一部の回答を短くしてみてください。

X26) REB/IRB Approval and Ethical Considerations [recommended as subheading under "Methods"] (not a CONSORT item)

X26-i) Comment on ethics committee approval

	1	2	3	4	5	
subitem not at all important	○	○	○	○	○	essential

Does your paper address subitem X26-i?

Copy and paste relevant sections from the manuscript (include quotes in quotation marks "like this" to indicate direct quotes from your manuscript), or elaborate on this item by providing additional information not in the ms, or briefly explain why the item is not applicable/relevant for your study

"Ethics Approval
Ethics approval was given to the institutional review board of the First author's affiliation. This trial was registered with the University hospital Medical Information Network (UMIN) Center on the 16th of June (Registration number: UMIN000051354). "

x26-ii) Outline informed consent procedures

Outline informed consent procedures e.g., if consent was obtained offline or online (how? Checkbox, etc.?), and what information was provided (see 4a-ii). See [6] for some items to be included in informed consent documents.

	1	2	3	4	5	
subitem not at all important	○	○	○	●	○	essential

選択を解除

回答のサイズが大きすぎます。一部の回答を短くしてみてください。

Does your paper address subitem X26-ii?

Copy and paste relevant sections from the manuscript (include quotes in quotation marks "like this" to indicate direct quotes from your manuscript), or elaborate on this item by providing additional information not in the ms, or briefly explain why the item is not applicable/relevant for your study

These advertisements directed potential participants to recruitment sites where participation information sheets and "informed consent were provided. "

X26-iii) Safety and security procedures

Safety and security procedures, incl. privacy considerations, and any steps taken to reduce the likelihood or detection of harm (e.g., education and training, availability of a hotline)

	1	2	3	4	5	
subitem not at all important	○	○	◉	○	○	essential

選択を解除

Does your paper address subitem X26-iii?

Copy and paste relevant sections from the manuscript (include quotes in quotation marks "like this" to indicate direct quotes from your manuscript), or elaborate on this item by providing additional information not in the ms, or briefly explain why the item is not applicable/relevant for your study

"In this study, they were not informed about the conditions they were allocated to and the real nature of the study. Instead, they were informed that the study would examine the efficacy of the mHealth app (Intellect) in promoting well-being. "

RESULTS

13a) For each group, the numbers of participants who were randomly assigned, received intended treatment, and were analysed for the primary outcome

NPT: The number of care providers or centers performing the intervention in each group

回答のサイズが大きすぎます。一部の回答を短くしてみてください。

Does your paper address CONSORT subitem 13a? *

Copy and paste relevant sections from the manuscript (include quotes in quotation marks "like this" to indicate direct quotes from your manuscript), or elaborate on this item by providing additional information not in the ms, or briefly explain why the item is not applicable/relevant for your study

"Participant Flow, Characteristics, and Recruitment
Of the 73 participants, 47.94% (n = 35, mean age = 38.91 years, SD = 10.03) and 52.05% (n = 38, mean age = 41.82 years, SD = 10.80) were assigned to INTELLECT and AO groups, respectively. Of the 35 participants allocated to the INTELLECT group, 8 were non-responsive at the 2-month follow-up, as were 4 of the 38 participants in the AO group (Figure 2)." The study period spanned between June 16th, 2023 and March 31st, 2024. Tables 1 shows participants' demographic data.

Group differences for the Primary Outcome Variable
"72 participants were included in the analysis. " The results showed that participants' PHQ-4 scores significantly decreased from baseline at both post-intervention and 2-month follow-up. There was a significant interaction between group and time at post-intervention ($t(364.7426) = -2.243$, $p < .05$) and 2-month follow-up ($t(364.6948) = -3.284$, $p < .05$). As shown in Table 2, the effect sizes were moderate and large at post-intervention and follow-up respectively ((Cohen's d = -.57, 95% CI = -1.07 to -0.06)) at post-intervention; (Cohen's d = -.85, 95% CI = -1.38 to -0.32) at 2-month follow-up).

13b) For each group, losses and exclusions after randomisation, together with reasons

Does your paper address CONSORT subitem 13b? (NOTE: Preferably, this is shown in a CONSORT flow diagram) *

Copy and paste relevant sections from the manuscript (include quotes in quotation marks "like this" to indicate direct quotes from your manuscript), or elaborate on this item by providing additional information not in the ms, or briefly explain why the item is not applicable/relevant for your study

Of the 73 participants, 47.94% (n = 35, mean age = 38.91 years, SD = 10.03) and 52.05% (n = 38, mean age = 41.82 years, SD = 10.80) were assigned to INTELLECT and AO groups, respectively. Of the 35 participants allocated to the INTELLECT group, 8 were non-responsive at the 2-month follow-up, as were 4 of the 38 participants in the AO group ("Figure 2").

回答のサイズが大きすぎます。一部の回答を短くしてみてください。

13b-i) Attrition diagram

Strongly recommended: An attrition diagram (e.g., proportion of participants still logging in or using the intervention/comparator in each group plotted over time, similar to a survival curve) or other figures or tables demonstrating usage/dose/engagement.

	1	2	3	4	5	
subitem not at all important	○	○	○	○	○	essential

Does your paper address subitem 13b-i?

Copy and paste relevant sections from the manuscript or cite the figure number if applicable (include quotes in quotation marks "like this" to indicate direct quotes from your manuscript), or elaborate on this item by providing additional information not in the ms, or briefly explain why the item is not applicable/relevant for your study

Participant Flow, Characteristics, and Recruitment
Of the 73 participants, 47.94% (n = 35, mean age = 38.91 years, SD = 10.03) and 52.05% (n = 38, mean age = 41.82 years, SD = 10.80) were assigned to INTELLECT and AO groups, respectively. "Of the 35 participants allocated to the INTELLECT group, 8 were non-responsive at the 2-month follow-up, as were 4 of the 38 participants in the AO group (Figure 2)." " The study period spanned between June 16th, 2023 and March 31st, 2024. Tables 1 shows participants' demographic data.

14a) Dates defining the periods of recruitment and follow-up

Does your paper address CONSORT subitem 14a? *

Copy and paste relevant sections from the manuscript (include quotes in quotation marks "like this" to indicate direct quotes from your manuscript), or elaborate on this item by providing additional information not in the ms, or briefly explain why the item is not applicable/relevant for your study

"The study period spanned between June 16th, 2023 and March 31st, 2024."

回答のサイズが大きすぎます。一部の回答を短くしてみてください。

14a-i) Indicate if critical “secular events” fell into the study period

Indicate if critical “secular events” fell into the study period, e.g., significant changes in Internet resources available or “changes in computer hardware or Internet delivery resources”

	1	2	3	4	5	
subitem not at all important	◉	○	○	○	○	essential

選択を解除

Does your paper address subitem 14a-i?

Copy and paste relevant sections from the manuscript (include quotes in quotation marks "like this" to indicate direct quotes from your manuscript), or elaborate on this item by providing additional information not in the ms, or briefly explain why the item is not applicable/relevant for your study

this is not applicable in this study

14b) Why the trial ended or was stopped (early)

Does your paper address CONSORT subitem 14b? *

Copy and paste relevant sections from the manuscript (include quotes in quotation marks "like this" to indicate direct quotes from your manuscript), or elaborate on this item by providing additional information not in the ms, or briefly explain why the item is not applicable/relevant for your study

This is not applicable in this study

15) A table showing baseline demographic and clinical characteristics for each group

NPT: When applicable, a description of care providers (case volume, qualification, expertise, etc.) and centers (volume) in each group

回答のサイズが大きすぎます。一部の回答を短くしてみてください。

Does your paper address CONSORT subitem 15? *

Copy and paste relevant sections from the manuscript (include quotes in quotation marks "like this" to indicate direct quotes from your manuscript), or elaborate on this item by providing additional information not in the ms, or briefly explain why the item is not applicable/relevant for your study

"Table 1 Participant demographics and clinical characters (n = 73) "

15-i) Report demographics associated with digital divide issues

In ehealth trials it is particularly important to report demographics associated with digital divide issues, such as age, education, gender, social-economic status, computer/Internet/ehealth literacy of the participants, if known.

	1	2	3	4	5	
subitem not at all important	○	○	○	○	◉	essential

選択を解除

Does your paper address subitem 15-i? *

Copy and paste relevant sections from the manuscript (include quotes in quotation marks "like this" to indicate direct quotes from your manuscript), or elaborate on this item by providing additional information not in the ms, or briefly explain why the item is not applicable/relevant for your study

"Table 1 Participant demographics and clinical characters (n = 73) "

16) For each group, number of participants (denominator) included in each analysis and whether the analysis was by original assigned groups

回答のサイズが大きすぎます。一部の回答を短くしてみてください。

16-i) Report multiple “denominators” and provide definitions

Report multiple “denominators” and provide definitions: Report N’s (and effect sizes) “across a range of study participation [and use] thresholds” [1], e.g., N exposed, N consented, N used more than x times, N used more than y weeks, N participants “used” the intervention/comparator at specific pre-defined time points of interest (in absolute and relative numbers per group). Always clearly define “use” of the intervention.

	1	2	3	4	5	
subitem not at all important	○	○	○	○	○	essential

Does your paper address subitem 16-i? *

Copy and paste relevant sections from the manuscript (include quotes in quotation marks "like this" to indicate direct quotes from your manuscript), or elaborate on this item by providing additional information not in the ms, or briefly explain why the item is not applicable/relevant for your study

Participant Flow, Characteristics, and Recruitment
Of the 73 participants, 47.94% (n = 35, mean age = 38.91 years, SD = 10.03) and 52.05% (n = 38, mean age = 41.82 years, SD = 10.80) were assigned to INTELLECT and AO groups, respectively. Of the 35 participants allocated to the INTELLECT group, 8 were non-responsive at the 2-month follow-up, as were 4 of the 38 participants in the AO group ("Figure 2").

16-ii) Primary analysis should be intent-to-treat

Primary analysis should be intent-to-treat, secondary analyses could include comparing only “users”, with the appropriate caveats that this is no longer a randomized sample (see 18-i).

	1	2	3	4	5	
subitem not at all important	◉	○	○	○	○	essential

選択を解除

回答のサイズが大きすぎます。一部の回答を短くしてみてください。

Does your paper address subitem 16-ii?

Copy and paste relevant sections from the manuscript (include quotes in quotation marks "like this" to indicate direct quotes from your manuscript), or elaborate on this item by providing additional information not in the ms, or briefly explain why the item is not applicable/relevant for your study

"We used linear mixed modelling (LMM) to analyze the study's primary outcome measures. LMM was selected because of its strength in accommodating missing data" and its ability to incorporate random effects into analyses.

17a) For each primary and secondary outcome, results for each group, and the estimated effect size and its precision (such as 95% confidence interval)

Does your paper address CONSORT subitem 17a? *

Copy and paste relevant sections from the manuscript (include quotes in quotation marks "like this" to indicate direct quotes from your manuscript), or elaborate on this item by providing additional information not in the ms, or briefly explain why the item is not applicable/relevant for your study

"Group differences for the Primary Outcome Variable
72 participants were included in the analysis. The results showed that participants' PHQ-4 scores significantly decreased from baseline at both post-intervention and 2-month follow-up. There was a significant interaction between group and time at post-intervention (t (364.7426) = −2.243, $p < .05$) and 2-month follow-up (t (364.6948) = −3.284, $p < .05$). As shown in Table 2, the effect sizes were moderate and large at post-intervention and follow-up respectively ((Cohen's d = -.57, 95% CI = -1.07 to -0.06)) at post-intervention; (Cohen's d = -.85, 95% CI = -1.38 to -0.32) at 2-month follow-up)."

回答のサイズが大きすぎます。一部の回答を短くしてみてください。

17a-i) Presentation of process outcomes such as metrics of use and intensity of use

In addition to primary/secondary (clinical) outcomes, the presentation of process outcomes such as metrics of use and intensity of use (dose, exposure) and their operational definitions is critical. This does not only refer to metrics of attrition (13-b) (often a binary variable), but also to more continuous exposure metrics such as "average session length". These must be accompanied by a technical description how a metric like a "session" is defined (e.g., timeout after idle time) [1] (report under item 6a).

	1	2	3	4	5	
subitem not at all important	○	○	○	○	◉	essential

選択を解除

Does your paper address subitem 17a-i?

Copy and paste relevant sections from the manuscript (include quotes in quotation marks "like this" to indicate direct quotes from your manuscript), or elaborate on this item by providing additional information not in the ms, or briefly explain why the item is not applicable/relevant for your study

"Group differences for the Secondary Variable
As shown in Table 3, the results showed that participants' CBTSS scores increased post-intervention and follow-up. The intervention group showed a significant improvement in CBTSS's self-monitoring score only at post-intervention compared to the control group (t (120.7526) = 2.672, p < .05). The effect size at post-intervention was moderate (Cohen's d = 0.68, 95% CI = 0.17 to 1.18).
"

17b) For binary outcomes, presentation of both absolute and relative effect sizes is recommended

回答のサイズが大きすぎます。一部の回答を短くしてみてください。

Does your paper address CONSORT subitem 17b? *

Copy and paste relevant sections from the manuscript (include quotes in quotation marks "like this" to indicate direct quotes from your manuscript), or elaborate on this item by providing additional information not in the ms, or briefly explain why the item is not applicable/relevant for your study

this is not applicable in this study

18) Results of any other analyses performed, including subgroup analyses and adjusted analyses, distinguishing pre-specified from exploratory

Does your paper address CONSORT subitem 18? *

Copy and paste relevant sections from the manuscript (include quotes in quotation marks "like this" to indicate direct quotes from your manuscript), or elaborate on this item by providing additional information not in the ms, or briefly explain why the item is not applicable/relevant for your study

this is not applicable

18-i) Subgroup analysis of comparing only users

A subgroup analysis of comparing only users is not uncommon in ehealth trials, but if done, it must be stressed that this is a self-selected sample and no longer an unbiased sample from a randomized trial (see 16-iii).

	1	2	3	4	5	
subitem not at all important	◉	○	○	○	○	essential

選択を解除

回答のサイズが大きすぎます。一部の回答を短くしてみてください。

Does your paper address subitem 18-i?

Copy and paste relevant sections from the manuscript (include quotes in quotation marks "like this" to indicate direct quotes from your manuscript), or elaborate on this item by providing additional information not in the ms, or briefly explain why the item is not applicable/relevant for your study

this is not applicable

19) All important harms or unintended effects in each group
(for specific guidance see CONSORT for harms)

Does your paper address CONSORT subitem 19? *

Copy and paste relevant sections from the manuscript (include quotes in quotation marks "like this" to indicate direct quotes from your manuscript), or elaborate on this item by providing additional information not in the ms, or briefly explain why the item is not applicable/relevant for your study

we did not collect important harms

19-i) Include privacy breaches, technical problems

Include privacy breaches, technical problems. This does not only include physical “harm” to participants, but also incidents such as perceived or real privacy breaches [1], technical problems, and other unexpected/unintended incidents. “Unintended effects” also includes unintended positive effects [2].

	1	2	3	4	5	
subitem not at all important	(●)	○	○	○	○	essential

選択を解除

回答のサイズが大きすぎます。一部の回答を短くしてみてください。

Does your paper address subitem 19-i?

Copy and paste relevant sections from the manuscript (include quotes in quotation marks "like this" to indicate direct quotes from your manuscript), or elaborate on this item by providing additional information not in the ms, or briefly explain why the item is not applicable/relevant for your study

this is not applicable in our study

19-ii) Include qualitative feedback from participants or observations from staff/researchers

Include qualitative feedback from participants or observations from staff/researchers, if available, on strengths and shortcomings of the application, especially if they point to unintended/unexpected effects or uses. This includes (if available) reasons for why people did or did not use the application as intended by the developers.

	1	2	3	4	5	
subitem not at all important	○	○	○	○	◉	essential

選択を解除

回答のサイズが大きすぎます。一部の回答を短くしてみてください。

Does your paper address subitem 19-ii?

Copy and paste relevant sections from the manuscript (include quotes in quotation marks "like this" to indicate direct quotes from your manuscript), or elaborate on this item by providing additional information not in the ms, or briefly explain why the item is not applicable/relevant for your study

"App engagement among the intervention group
As shown in Table 4, the average degree of satisfaction measured by the iOSDMH among users of the Intellect application was notably high. Around 16-24 (55.2%-82.8%) of the 29 intervention participants answered “agree” or “relatively agree” on each item of acceptability (n=16-24, 55.2%-82.8%), appropriateness (n=19-24, 65.5%-82.8%), feasibility (n=7-23, 24.1%-79.3%). The highest proportions of positive responses in each of the 3 aspects were “ this program is acceptable for me”(n=24, 82.8%) in acceptability, “the content of the program is appropriate” (n=24, 82.8%) in appropriateness, and “the length of 1 content is implementable; the program is easy to understand” (n=23, 79.3) in feasibility, respectively. Regarding overall satisfaction with Intellect app, 23 (79.3%) participants answered “agree” or “relatively agree”. Regarding harms, 2-8 (6.8%-27.5.4%) participants answered “agree” or “relatively agree”, except for “excessive pressure on learning this program regularly” (n=12, 41.4%). An examination of the engagement with individual components over a four-week period revealed that the 'Wellbeing Check-in' module was the most frequently utilized, followed in descending order of usage by the 'Rescue Sessions,' and 'Guided Journals'. Notably, the 'Learning Path' component required the most significant investment of time from users. This substantial time requirement for the 'Learning Path' is likely the reason for the observed discrepancy between the number of initial engagements with the component and the lower rate of completions."

DISCUSSION

22) Interpretation consistent with results, balancing benefits and harms, and considering other relevant evidence

NPT: In addition, take into account the choice of the comparator, lack of or partial blinding, and unequal expertise of care providers or centers in each group

回答のサイズが大きすぎます。一部の回答を短くしてみてください。

22-i) Restate study questions and summarize the answers suggested by the data, starting with primary outcomes and process outcomes (use)

Restate study questions and summarize the answers suggested by the data, starting with primary outcomes and process outcomes (use).

	1	2	3	4	5	
subitem not at all important	○	○	○	○	◉	essential

選択を解除

回答のサイズが大きすぎます。一部の回答を短くしてみてください。

Does your paper address subitem 22-i? *

Copy and paste relevant sections from the manuscript (include quotes in quotation marks "like this" to indicate direct quotes from your manuscript), or elaborate on this item by providing additional information not in the ms, or briefly explain why the item is not applicable/relevant for your study

回答のサイズが大きすぎます。一部の回答を短くしてみてください。

Our primary hypothesis was supported, with the intervention group showing significantly greater improvements in depression and anxiety levels at post-intervention and 2-month follow-up. Effect sizes of these improvements at each timepoint were considerably larger than those reported in similar studies reviewed by a recent meta-analysis (Firth et al., 2017). These findings add to the scant literature by showing that self-guided CBT features can be highly effective in reducing anxiety and depressive symptoms among clinically at-risk working adults in Japan. This aligns with previous research showing comparable efficacy of brief CBT-based mobile features, or computerized CBT in alleviating depressive symptoms among university students experiencing baseline depressive symptoms in Western societies (Mantani et al., 2017; Bruhns et al., 2021; Six et al., 2022).

Our secondary hypothesis was partially supported. The self-guided CBT features also demonstrated significantly greater improvements in the self-monitoring aspect of cognitive behavioural skills than the waitlist control group, as assessed through the CBTSS. The effect size of this improvement at post-intervention was moderate (Cohen's d = .68, 95% CI = 0.17 to 1.18). Self-monitoring questions are prominently featured throughout INTELLECT's self-guided features. For example, thought records are embedded within all of INTELLECT's "Learning Paths" "Rescue Sessions" and "Guided Journals," consistently prompting each participant to analyze their own thought patterns. It is probable that the decrease in anxiety and depressive symptoms among our participants was related to the increase in self-monitoring. This technique alone has previously shown efficacy in alleviating anxiety and depressive symptoms in adults (Rickard et al., 2016; Bakker and Rickard, 2018) and youths diagnosed with major depression (Hetrick et al., 2018). However, our study did not conduct longitudinal mediation analyses to confirm this hypothesis. Future research could utilize more rigorous statistical methods, such as multilevel mediation analyses to support this claim. In contrast, for other skills assessed by the CBTSS (i.e., Behavioral Activation, Assertiveness Communication, Problem Solving), only specific self-guided features within INTELLECT were tailored to enhance these skills. For example, within INTELLECT's suite of Learning Paths, approximately half of them concentrate on modifying behavioral patterns, while only three focus on modifying communication styles. Researchers have also proposed that the anticipated type of improvements resulting from the interventions correlate with the type of content offered within the intervention (Morrison et al. (2012)). Future researchers are encouraged to isolate various types of self-guided interventions and assess the efficacy of each intervention using a similar RCT design to ours.. In comparison to the control group, the intervention group also did not exhibit significantly greater improvements in the cognitive restructuring aspect of CBT skills. There are a couple of possible reasons. Firstly, cognitive restructuring has been identified as one of the most challenging CBT skills to impart, even among trained counselors and psychotherapists with many years of experience (Stark et al., 2011). Furthermore, cognitive restructuring is widely recognized as a difficult skill to master independently, both within and outside of therapeutic contexts (Willner et al., 2005; Neal-Barnett et al., 2019). Further research is needed to explore suitable methodologies for effectively teaching cognitive restructuring skills within self-help modalities, enabling users to learn autonomously. Finally, it is important to note that participants in both the intervention and control group demonstrated significant improvements in the CBT skills across time (see Table 3). This particular control group may have masked the intervention effects on CBT skills. Future researchers can more effectively assess the efficacy of INTELLECT's self-guided CBT interventions by employing a three-armed randomized controlled trial (RCT), wherein the efficacy of these CBT features is compared against those of an active control group and a waitlist control group.

回答のサイズが大きすぎます。一部の回答を短くしてみてください。

22-ii) Highlight unanswered new questions, suggest future research

Highlight unanswered new questions, suggest future research.

	1	2	3	4	5	
subitem not at all important	○	○	○	○	◉	essential

選択を解除

Does your paper address subitem 22-ii?

Copy and paste relevant sections from the manuscript (include quotes in quotation marks "like this" to indicate direct quotes from your manuscript), or elaborate on this item by providing additional information not in the ms, or briefly explain why the item is not applicable/relevant for your study

"These findings add to the scant literature by showing that self-guided CBT features can be highly effective in reducing anxiety and depressive symptoms among clinically at-risk working adults in Japan. "

"Finally, it is noteworthy that a sizable percentage of our participants expressed satisfaction with using INTELLECT, rating it highly in terms of acceptability, appropriateness for improving mental health, and ease of understanding. The self-help features of INTELLECT, which utilize plain language, illustrations, videos, and audio narration to explain evidence-based content, may contribute to these positive implementation outcomes. Given the substantial effect sizes and user-friendly design of this application, we recommend broader dissemination of the INTELLECT app to alleviate anxiety and depressive symptoms among employees in need. "

20) Trial limitations, addressing sources of potential bias, imprecision, and, if relevant, multiplicity of analyses

回答のサイズが大きすぎます。一部の回答を短くしてみてください。

20-i) Typical limitations in ehealth trials

Typical limitations in ehealth trials: Participants in ehealth trials are rarely blinded. Ehealth trials often look at a multiplicity of outcomes, increasing risk for a Type I error. Discuss biases due to non-use of the intervention/usability issues, biases through informed consent procedures, unexpected events.

	1	2	3	4	5	
subitem not at all important	○	○	○	○	◉	essential

選択を解除

Does your paper address subitem 20-i? *

Copy and paste relevant sections from the manuscript (include quotes in quotation marks "like this" to indicate direct quotes from your manuscript), or elaborate on this item by providing additional information not in the ms, or briefly explain why the item is not applicable/relevant for your study

Limitation
This study has three limitations. Firstly,

"although INTELLECT’s self-help interventions significantly reduced depressive symptoms during the study period and at the 2-month follow-up, it is uncertain if these improvements would persist beyond two months".

Previous research on similar internet-based CBT interventions suggests a potential decline in efficacy over extended durations. Hence, it is important for future studies to evaluate the longitudinal effects of mobile-based CBT interventions. Secondly,

"this intervention study primarily focused on assessing improvements in depressive symptoms and user engagement with the application, overlooking potential adverse effects associated with its use."

A thorough examination of post-intervention side effects is essential to ensure the app's safety across diverse user demographics. Lastly,

"while the INTELLECT app effectively enhanced self-monitoring capabilities, it did not significantly influence the development of other crucial CBT-related skills, such as cognitive restructuring, behavioral activation, communication, or problem-solving."

Future studies may explore strategies for enhancing the application to facilitate a more comprehensive development of these skills, which could augment INTELLECT’s efficacy in addressing anxiety and depressive symptoms.

回答のサイズが大きすぎます。一部の回答を短くしてみてください。

21) Generalisability (external validity, applicability) of the trial findings
NPT: External validity of the trial findings according to the intervention, comparators, patients, and care providers or centers involved in the trial

21-i) Generalizability to other populations
Generalizability to other populations: In particular, discuss generalizability to a general Internet population, outside of a RCT setting, and general patient population, including applicability of the study results for other organizations

	1	2	3	4	5	
subitem not at all important	○	○	○	◉	○	essential

選択を解除

Does your paper address subitem 21-i?
Copy and paste relevant sections from the manuscript (include quotes in quotation marks "like this" to indicate direct quotes from your manuscript), or elaborate on this item by providing additional information not in the ms, or briefly explain why the item is not applicable/relevant for your study

"This study provides the first evidence of the efficacy of the INTELLECT app including all components in reducing depressive symptoms among Japanese adult workers. "

21-ii) Discuss if there were elements in the RCT that would be different in a routine application setting
Discuss if there were elements in the RCT that would be different in a routine application setting (e.g., prompts/reminders, more human involvement, training sessions or other co-interventions) and what impact the omission of these elements could have on use, adoption, or outcomes if the intervention is applied outside of a RCT setting.

	1	2	3	4	5	
subitem not at all important	○	○	○	○	○	essential

回答のサイズが大きすぎます。一部の回答を短くしてみてください。

Does your paper address subitem 21-ii?

Copy and paste relevant sections from the manuscript (include quotes in quotation marks "like this" to indicate direct quotes from your manuscript), or elaborate on this item by providing additional information not in the ms, or briefly explain why the item is not applicable/relevant for your study

Firstly, although INTELLECT's self-help interventions significantly reduced depressive symptoms during the study period and at the 2-month follow-up, it is uncertain if these improvements would persist beyond two months. "Previous research on similar internet-based CBT interventions suggests a potential decline in efficacy over extended durations. Hence, it is important for future studies to evaluate the longitudinal effects of mobile-based CBT interventions. "

OTHER INFORMATION

23) Registration number and name of trial registry

Does your paper address CONSORT subitem 23? *

Copy and paste relevant sections from the manuscript (include quotes in quotation marks "like this" to indicate direct quotes from your manuscript), or elaborate on this item by providing additional information not in the ms, or briefly explain why the item is not applicable/relevant for your study

"This trial was registered with the University hospital Medical Information Network (UMIN) Center on the 16th of June (Registration number: UMIN000051354). "

24) Where the full trial protocol can be accessed, if available

回答のサイズが大きすぎます。一部の回答を短くしてみてください。

Does your paper address CONSORT subitem 24? *

Cite a Multimedia Appendix, other reference, or copy and paste relevant sections from the manuscript (include quotes in quotation marks "like this" to indicate direct quotes from your manuscript), or elaborate on this item by providing additional information not in the ms, or briefly explain why the item is not applicable/relevant for your study

回答を入力

25) Sources of funding and other support (such as supply of drugs), role of funders

Does your paper address CONSORT subitem 25? *

Copy and paste relevant sections from the manuscript (include quotes in quotation marks "like this" to indicate direct quotes from your manuscript), or elaborate on this item by providing additional information not in the ms, or briefly explain why the item is not applicable/relevant for your study

Acknowledgements
"This study was self-funded by Intellect Pte Ltd "under a Research Collaboration Agreement with the University of Human Environments, Japan.

X27) Conflicts of Interest (not a CONSORT item)

X27-i) State the relation of the study team towards the system being evaluated

In addition to the usual declaration of interests (financial or otherwise), also state the relation of the study team towards the system being evaluated, i.e., state if the authors/evaluators are distinct from or identical with the developers/sponsors of the intervention.

	1	2	3	4	5	
subitem not at all important	○	○	○	○	◉	essential

選択を解除

回答のサイズが大きすぎます。一部の回答を短くしてみてください。

Does your paper address subitem X27-i?

Copy and paste relevant sections from the manuscript (include quotes in quotation marks "like this" to indicate direct quotes from your manuscript), or elaborate on this item by providing additional information not in the ms, or briefly explain why the item is not applicable/relevant for your study

"Conflicts of Interest
The study was fully funded by Intellect Pte Ltd. The study design, data management, interpretation, analysis, reporting, and decision to publish of the study are entirely independent of Intellect Pte Ltd."

About the CONSORT EHEALTH checklist

As a result of using this checklist, did you make changes in your manuscript? *

- ○ yes, major changes
- ◉ yes, minor changes
- ○ no

What were the most important changes you made as a result of using this checklist?

回答を入力

How much time did you spend on going through the checklist INCLUDING making changes in your manuscript *

I spent on modifying for a few hours

回答のサイズが大きすぎます。一部の回答を短くしてみてください。

As a result of using this checklist, do you think your manuscript has improved? *

- ◯ yes
- ◯ no
- ◉ その他: Yes, but because all members do not understand this checklist,

Would you like to become involved in the CONSORT EHEALTH group?

This would involve for example becoming involved in participating in a workshop and writing an "Explanation and Elaboration" document

- ◯ yes
- ◉ no
- ◯ その他:

選択を解除

Any other comments or questions on CONSORT EHEALTH

回答を入力

STOP - Save this form as PDF before you click submit

To generate a record that you filled in this form, we recommend to generate a PDF of this page (on a Mac, simply select "print" and then select "print as PDF") before you submit it.

When you submit your (revised) paper to JMIR, please upload the PDF as supplementary file.

Don't worry if some text in the textboxes is cut off, as we still have the complete information in our database. Thank you!

Final step: Click submit !

[illegible]

回答のサイズが大きすぎます。一部の回答を短くしてみてください。

送信　　フォームをクリア

Google フォームでパスワードを送信しないでください。

このコンテンツは Google が作成または承認したものではありません。 不正行為の報告 - 利用規約 - プライバシーポリシー

Google フォーム

回答のサイズが大きすぎます。一部の回答を短くしてみてください。

回答のサイズが大きすぎます。一部の回答を短くしてみてください。
